# Electric Field Tunability of Photoluminescence from a Hybrid Peptide–Plasmonic Metal Microfabricated Chip

**DOI:** 10.1021/jacsau.1c00323

**Published:** 2021-10-08

**Authors:** Sawsan Almohammed, Okan K. Orhan, Sorcha Daly, David D. O’Regan, Brian J. Rodriguez, Eoin Casey, James H. Rice

**Affiliations:** † School of Physics, University College Dublin, Belfield, Dublin D04 V1W8, Ireland; ‡ Conway Institute of Biomolecular and Biomedical Research, University College Dublin, Belfield, Dublin D04 V1W8, Ireland; § School of Physics, AMBER, and CRANN Institute, Trinity College Dublin, The University of Dublin, Dublin D02 PN40, Ireland; ∥ School of Chemical and Bioprocess Engineering, University College Dublin, Belfield, Dublin D04 V1W8, Ireland

**Keywords:** diphenylalanine peptide nanotubes, self-assembly, *Pseudomonas fluorescens*, bovine serum albumin, surface-enhanced fluorescence

## Abstract

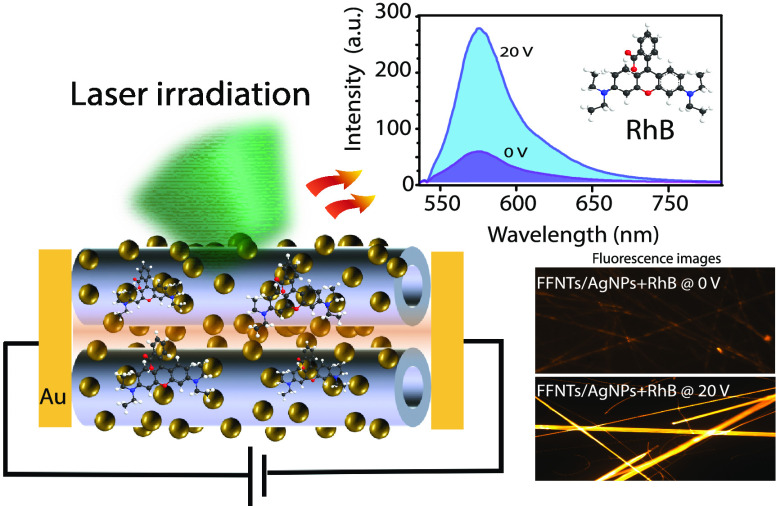

Enhancement of fluorescence through the application of plasmonic metal nanostructures has gained substantial research attention due to the widespread use of fluorescence-based measurements and devices. Using a microfabricated plasmonic silver nanoparticle–organic semiconductor platform, we show experimentally the enhancement of fluorescence intensity achieved through electro-optical synergy. Fluorophores located sufficiently near silver nanoparticles are combined with diphenylalanine nanotubes (FFNTs) and subjected to a DC electric field. It is proposed that the enhancement of the fluorescence signal arises from the application of the electric field along the length of the FFNTs, which stimulates the pairing of low-energy electrons in the FFNTs with the silver nanoparticles, enabling charge transport across the metal–semiconductor template that enhances the electromagnetic field of the plasmonic nanoparticles. Many-body perturbation theory calculations indicate that, furthermore, the charging of silver may enhance its plasmonic performance intrinsically at particular wavelengths, through band-structure effects. These studies demonstrate for the first time that field-activated plasmonic hybrid platforms can improve fluorescence-based detection beyond using plasmonic nanoparticles alone. In order to widen the use of this hybrid platform, we have applied it to enhance fluorescence from bovine serum albumin and *Pseudomonas fluorescens*. Significant enhancement in fluorescence intensity was observed from both. The results obtained can provide a reference to be used in the development of biochemical sensors based on surface-enhanced fluorescence.

## Introduction

Plasmonic enhanced fluorescence (PEF) is produced through effective coupling of a metal nanostructure localized surface plasmon resonance (LSPR) with photoexcited fluorophores to increase their photoluminescence signal intensity. PEF can be applied to improve the performance of fluorescence-based measurements in the fields of chemistry,^1,2^ biology,^3,4^ materials,^5,6^ photonics,^1,7^ and medicine.^8,9^ Metal nanoparticles are particularly useful for PEF applications, as their LSPRs^7,10,11^ can be adjusted by their geometry^7,10,11^ in addition to the application of an external electric field, in combination with semiconductor materials, as reported previously.^12,13^

It is generally accepted that PEF emission occurs from local electromagnetic field enhancement or increased quantum yield, allied with a rise in the radiative decay rate.^14,15^ The interaction between the fluorophore and a metallic surface can result in the reduction of fluorescence when the fluorophore is separated from the metal surface by less than a few nanometers,^10,16−19^ and yet enhanced fluorescence has also been observed for fluorophores in contact with silver nanoparticles (AgNPs).^20^ A number of techniques have been used to make PEF emission substrates, including top-down lithographic methods including e-beam lithography,^10,14,21^ colloidal lithography,^22^ vacuum evaporation,^23^ and nanoimprinting.^24^ These techniques generally have a limitation, either high cost or lack of reproducibility or sensitivity. Bottom-up fabrication methods based on self-assembly, however, have proved to be a reproducible, highly sensitive method for PEF substrate fabrication.^10,14,21,25^

Peptide-based nanofabrication is an appealing method for making nanomaterials, given the use of peptides for molecular recognition and their elevated thermal and chemical stability,^26,27^ as well as their easy chemical preparation that does not require heavy metals, strong acids, or harsh chemical substances.^26,27^ Among the numerous peptide-molecule-based nanostructures, diphenylalanine (Phe-Phe, FF) is a frequently used building-block peptide that can form various structures such as nanotubes through self-assembly processes.^28,29^ Diphenylalanine can self-assemble into various morphologies, including nanotubes (FFNTs), which are wide-band-gap semiconductor organic materials.^30^ FFNTs also show chemical and thermal stability^31^ and mechanical strength,^32^ additional features required for optical, electrical, and biological applications.^33^ Several studies have shown the application of a plasmonic–semiconductor template formed from aligned FFNTs to enhance both chemical reactions and surface-enhanced scattering intensity, accomplished through electro-optical synergy.^12,34,35^ Here, we demonstrate for the first time the enhancement of fluorescence intensity via field-activated microfabricated plasmonic nanostructures combined with an organic FFNT template. We show that this optoelectrical device significantly boosts the PEF emission from a range of systems, including molecules, nanocrystals, proteins, and bacteria, exploiting facile field-activated trans-template charge transfer processes and resulting in a strengthening of the plasmonic electromagnetic mechanism. This novel approach is adaptable and can be used on a range of plasmonic metal nanoparticle and semiconductor combinations. Because of its high sensitivity, this proof-of-principle microfabricated plasmonic template opens up the possibility to use such devices in early-stage disease diagnosis and biosensing applications.

## Results

**Figure 1:**
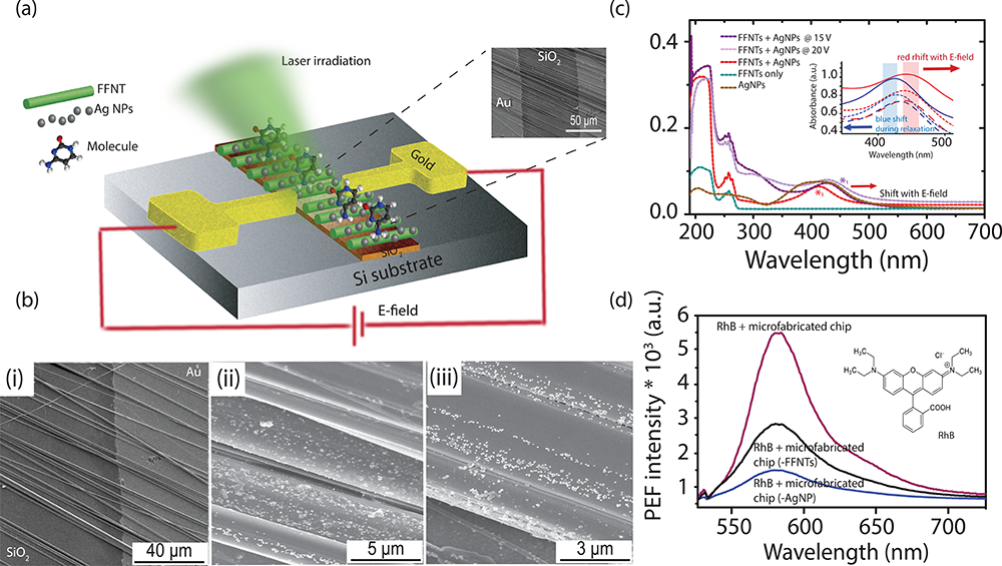
(a) Schematic illustration of the microfabricated chip device in air. The inset is a SEM image of the aligned FFNTs with AgNPs between gold electrodes. (b) SEM images of the aligned FFNTs and AgNPs, where (ii) and (iii) are the enlarged magnifications of (i). (c) Optical absorption UV–vis spectra of FFNTs with and without AgNPs (with and without electric field (*E* field)). The red asterisk (c) shows the LSPR of AgNPs at 420 nm (without *E* field), whereas the purple asterisk is for LSPR with *E* field (red-shifted to ∼435 nm). The dark purple spectrum is for an *E* field generated at 15 V, whereas the light purple spectrum is for an *E* field generated at 20 V. The inset in (c) shows red shifts of the LSPR of AgNPs with FFNTs with the application of electric field (red shading), followed by blue shifts during relaxation and the removal of the electric field (blue shading). (d) PEF and fluorescence spectra from RhB (schematic diagram of the molecule shown in inset) recorded on the microfabricated peptide semiconductor chip with and without AgNPs present.

A microfabricated chip design based on aligned FFNTs and AgNPs was prepared on Si substrates (shown schematically in Figure 1a), following a process reported previously.^12,13^ Gold electrodes were prepared on Si substrates, with aligned AgNP-decorated FFNTs formed between the electrodes. The inset in Figure 1a is a scanning electron microscopy (SEM) image of the aligned FFNTs between the gold electrodes, showing the FFNTs confined in the hydrophilic SiO_2_ region. SEM images (Figure 1b and Figure S1) show the alignment of the FFNTs and the morphology of the deposited AgNPs, which form along the FFNTs. The average distance between NPs is 30 ± 22 nm (*n* = 200 NPs) with a density of 152 ± 43 NPs/μm^2^, as determined from SEM images using ImageJ. In the optical absorption spectrum (Figure 1c) for the AgNPs alone it can be seen that the LSPR is located at ∼420 nm (full width at half-maximum (fwhm) of ∼45 nm). The absorption spectrum for FFNTs alone shows absorption peaks at ∼222 and ∼260 nm (Figure 1c), in agreement with previous reports.^36,37^ When FFNT is combined with AgNP, the metal LSPR feature is red-shifted by ∼16 nm (to ∼435 nm) and its fwhm narrowed by ∼15 nm (to ∼30 nm) (Figure 1c). This red shift and reduction in fwhm for the LSPR band of the metal nanoparticle has been reported to arise from the peptide amino acid carboxyl groups binding to metal NPs.^35,38,39^ The application of a DC electric field on the microfabricated chip device in air results in further shifts of the LSPR (∼20 nm shift) and broadening. The broadening and red shift in the LSPR of the AgNPs may result from aggregation and chemical interaction of the AgNPs on the FFNTs (Figure 1c), whereas the blue shift, during relaxation, can be understood as a result of an increased concentration of electron density in the Ag NPs (outlined in Figure 1c). Following the application of an external DC electric field (during relaxation), the change in the Ag NP electron density (Δ*N*/*N*) was calculated to be 9%, (where Δ*N*/*N* = 2Δλ/λ_0_, Δλ being the measured wavelength change and λ_0_ the original Ag NP plasmon peak position). This is similar to the electron density reported for a FFNT/Ag NP template following super-band-gap UV wavelength irradiation,^34,35^ which generated electrons that moved from the peptide to the metal nanoparticles as they reached charge equilibration.

The ability to manipulate or tune the LSPR of a metal using FFNTs and an applied electric field (red shift with the electric field on and blue shift with the electric field off (during relaxation)) could be advantageous for biochemical sensing and bioimaging applications.^35,38,39^

After optimization of the AgNP loading by which the NPs decorated and coated the FFNT (Figure S2), the fluorescence signal intensity from a fluorophore on the FFNT–AgNP plasmonic microfabricated chip was studied. The fluorescence from rhodamine B (RhB), widely used in biotechnology applications, was recorded with and without AgNPs present (Figure 1d). When AgNPs were present, a stronger fluorescence signal was recorded (Figure 1d). The enhanced fluorescence yield for RhB arose via PEF. The PEF intensity was higher when both FFNTs and AgNPs were present.

**Figure 2:**
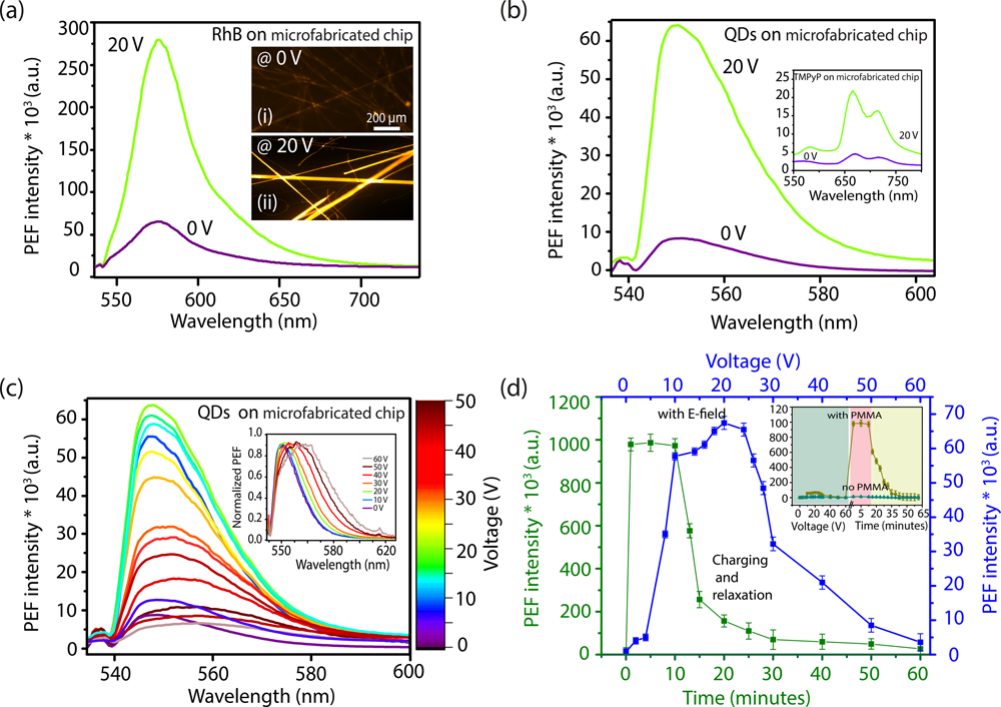
PEF emission spectra recorded for (a) RhB and (b) QD nanocrystal fluorophores with an applied electric field generated by the application of 20 V and with no applied field (0 V). The inset in (b) shows the corresponding spectrafor TMPyP. The inset in (a) shows fluorescence images recorded for FFNTs with RhB with (i) no electric field applied and (ii) with an electric field generated by the application of 20 V. (c) PEF emission spectra recorded for QD nanocrystals over a series of applied electric field strengths. The inset shows normalized spectra recorded for the QD nanocrystal fluorophores with variable applied electric field generated through the application of 0–60 V. (d) Plot of PEF emission spectral band position and fwhm with applied electric field strength (blue). Shown also is a plot of PEF signal intensity as a function of the applied voltage and the effect of the removal of electric field on the PEF signal intensity, as a function of time following removal of the electric field (green). The inset shows a plot of PEF intensity over electric field strength and then progression (relaxation) of the PEF signal after the electric field is removed.

Next, we investigated the influence of applying an electric field on the PEF emission intensity from a range of fluorophores (Figure 2), e.g., quantum dot (QD) nanocrystals and molecules RhB and *meso*-tetrakis(*N*-methyl-4-pyridyl)porphine tetrachloride (TMPyP), an important conjugated organic molecule that plays an essential role in the metabolism of living organisms. Techniques that can improve the efficiency or manipulate photoluminescence from QDs, RhB, or TMPyP could lead to lowering of the detection limits in medical assays. As indicated in the Introduction, the PEF mechanism depends on the separation distance between the metal NPs and the fluorophore. This distance dependence relates to the electromagnetic field intensity, which strongly decreases with distance and, following direct contact, can result in fluorescence quenching.^40,41^ Studies have shown that the addition of a thin polymer layer can introduce an approximately nanometer-thick distance between the analyte and the substrate, which can prevent quenching.^10,15^ For this reason, fluorophores at a concentration of ∼10^–9^ M were mixed with a dielectric polymer (PMMA) prior to PEF measurements and then deposited above the microfabricated chip device.

An electric field was applied to the hybrid template through the application of a DC voltage across the gold electrodes, from 0 to 60 V across a gap of ∼100 μm. The application of voltage in the <20 V range resulted in enhanced PEF emission intensity in comparison to 0 V for the three fluorophores studied (Figure 2a,b). The PEF emission signal reached a maximum when voltages from 20 to 30 V were applied. This is followed by a reduction in PEF emission intensity in the voltage range 30–60 V for all three fluorophores (Figure 2c,d and Figures S3–S5).

The enhancement in PEF emission signal potentially arises from an electric field activated charge transport mechanism, which strengthens the chemical enhancement in SERS. We previously reported an electric field activated induced coupling mechanism between low-energy electrons in the FFNTs with the AgNPs that led to charge moving across the template to the analyte molecules under study.^12^ Theoretical calculations showed that using a longitudinal electric field allowed the density of states of the FFNTs to be adjusted from a semiconductor to a near-metal (via a reduction in band gap). This supports efficient charge transfer from the FFNTs to the metal nanoparticles.^12^ The charge transfer processes are potentially optimized by aligning the peptide nanotubes. This in turn aligns the intrinsic electric dipoles of the FFNTs and maximizes the response of the FFNTs to the applied longitudinal electric field.

**Figure 3:**
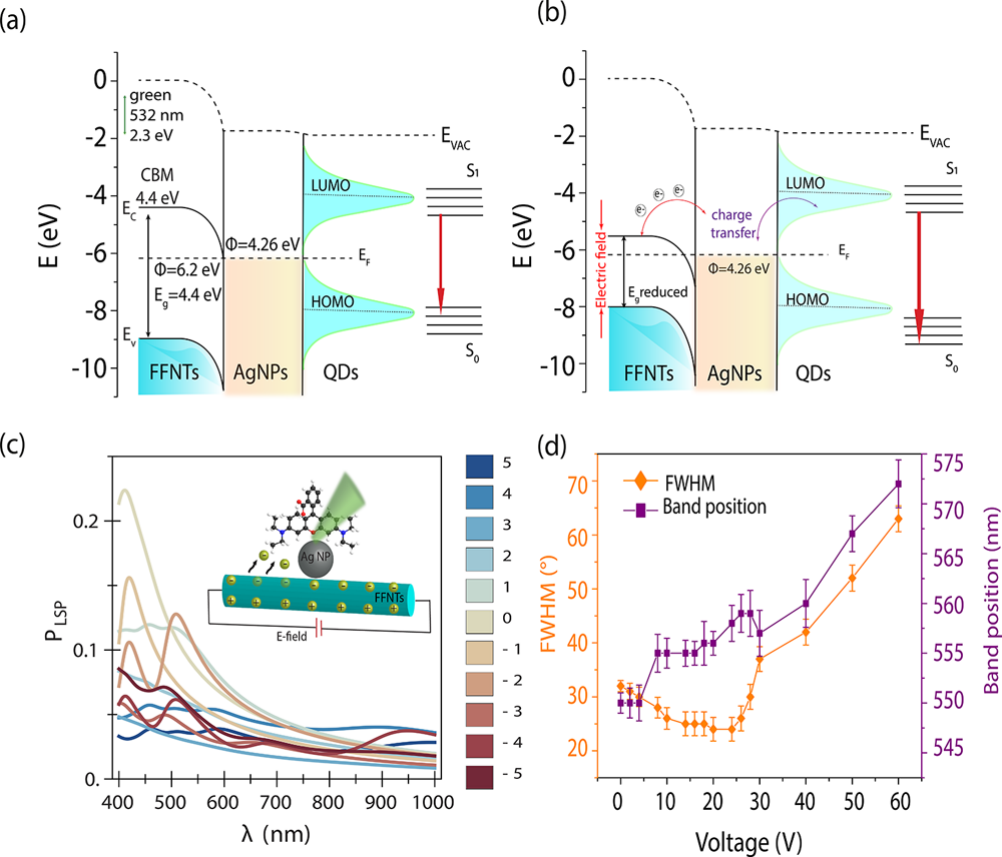
Band diagram of the FFNTs and AgNPs with QDs (a) before and (b) after an electric field is applied. S_0_ refers to the ground electronic state, and S_1_ is the lowest excited electronic state. (c) Calculations of the localized surface plasmon (LSP) frequency as a function of charge on Ag generated using first-principles many-body perturbation theory.^44^ Shown is a plot the wavelength-dependent localized-surface plasmon figure of merit (*P*_LSP_). The figure of merit arises from the product of the calculated electron energy loss spectra (EELS, used for plasmon generation) and quality factor (proportional to the lifetimes of the localized surface plasmons), for bulk Ag. Different curves show the results of charging Ag by percentages (legend bar) of up to ±5% with respect the neutral configuration valence electron count. (d) Plot of full width at half-maximum (fwhm) and band position of PEF spectra recorded over 0–60 V.

As shown schematically in Figure 3a,b, in the absence of an electric field, an FFNT is a semiconducting material with a band gap of ∼4.4 eV (as calculated from UV–vis data, Figure S6); however, following the application of an electric field, the band gap can be reduced to ∼3.8 eV (Figure S6), meaning that an FFNT becomes easily excited and strongly coupled with NPs, allowing efficient charge transfer from the FFNT to the metal NPs.^42^ Additionally, a resonance effect may contribute to the enhancement. Charge transfer resonance is related to photon-induced charge transfer from/to a semiconductor band edge to the electronic states of an adsorbed molecule.^42^ For instance, QDs with λ = 525 nm yield higher enhancement in comparison to QDs with λ = 665 nm; an ∼14-fold increase and an ∼8-fold increase, respectively, when a laser excitation wavelength of 532 nm is used (Figure S7). With the application of an electric field, the LSPR of the metal can be tuned to be close to the energy of the excitation laser to provide additional coupling with the probe molecules under study. According to the literature, the luminescence intensity could be further resonance-enhanced when the resonance peaks of metal NPs are matched with the luminescence peaks of QDs.^43^

Calculations of the quasi-particle band structure (as described in ref (44)) of the neutral and charged Ag (Figure 3c and Figures S8 and S9) were undertaken to simulate the effect of additional charge on the LSPR. The simulation of neutral Ag shows a strong localized-surface plasmon population (*P*_LSP_) at ∼410 nm (Figure 3c), in close agreement with experimental data (Figure 1c)). Negatively charging Ag shifts the primary plasmon peak spectrally to the red, being closer in resonance with the laser excitation frequency and strengthening electromagnetic enhancement of the fluorescence signal from the fluorophore. A 2% change in valence number produces the optimum generation of this secondary plasmon band. Adding a negative change that is too strong, such as a 5% change in valence number results, in a lowering of this secondary band intensity (Figure 3c). In contrast, positively charged Ag (Figure 3c and Figure S8) showed a substantial lowering of plasmonic generation.

Having only AgNPs, in the absence of FFNTs, did not result in an increase in PFE intensity. In contrast, a significant and continuous reduction in the PEF signal was observed with an applied electric field. This could be due to the occurrence of higher currents (Figure S10). To verify this, we measured the current in the substrate during the application of the electric field to be 118 mA at 60 V when only NPs were present. However, at the same voltage in the presence of FFNTs, a 26 mA current was measured. We have noticed that high currents lead to increased temperature. Metal NPs are easily oxidized in air, resulting in the lowering of both signal activity and signal reproducibility. FFNTs can passivate NPs and protect them from being oxidized at elevated temperatures due to the thermal and chemical stability of the FFNTs.^37^ Additionally, when only NPs are used, the PEF intensity does not return to its initial value during the relaxation period (after the electric field is turned off), suggesting that the presence of the FFNTs in the microfabricated chip is essential to preserve the signal integrity while both the electric field and high-power (∼60 mW) illumination are applied (Figure S11).

Accompanying the change in PEF emission intensity are changes in the fluorophores’ emission peak wavelength and line width. When we look first at the QD nanocrystal fluorophore, for instance, the PEF emission peak shifts λ_shift_ = 10 nm (from ∼550 to 560 nm) and the emission peak line width (λ_fwhm_ = 8 nm) shifts from 32 to 24 nm for fwhm when voltages of 0–20 V were applied (Figures 2c and 3d). This spectral red shift and peak narrowing correlating with an ∼15-fold strengthening of the PEF emission signal. Studies have reported that PEF emission spectra from QD nanocrystals are spectrally red shifted in addition to an enhancement in fluorescence signal strength when they are coupled with localized plasmons.^20^ Additionally, it has been reported that the red shift of the QD nanocrystal photoluminescence can be assigned to the exchanges between the transition dipole moments or electronic states of the QD nanocrystals and the dipole moment induced in the AgNPs,^20^ while the fluorescence emission fwhm of QD nanocrystal emitters is reduced as a result of the stronger interaction with localized plasmons.^20^ This is in agreement with theoretical calculations (Figure 2c, as outlined above), which showed that negatively charging the AgNP can create a new plasmon resonance frequency more in resonance with the QD emission frequency, leading to a stronger interaction of the QD nanocrystal emitter with localized plasmons. The red shift of the QD nanocrystal photoluminescence is attributed to the interactions between the permanent dipole moment of the QD nanocrystals and the dipole moment induced in the AgNPs.^20^ A similar change in the RhB fluorophore emission peak wavelength and line width was found (Figure S12). Relatively low quantum yield (QY) molecules at a concentration of 10^–8^ M were also investigated such as crystal violet (CV) (Figure S13), and a significant increase of ∼34-fold in PEF intensity was seen. Similarly to QDs, RhB, and TMPyP, changes in the emission peak wavelength and line width were also observed from those molecules. Fluorescence images (Figure S14) for the FFNTs (which are considered to be low-QY proteins) with and without an electric field also indicate the effectiveness of our approach to improve the PEF from low-QY materials.

Turning off the electric field resulted in an increase in PEF emission signal intensity from the QD nanocrystal fluorophore on the microfabricated chip. This intensity spike resulted in an ∼100-fold increase in PEF signal in comparison to the PEF signal recorded at 0 V (Figure 2d). This increased PEF signal intensity following removal of the external electric field persisted over ∼10 min before relaxing back to the original signal intensity observed before the electric field was applied (Figure 2d). The increased PEF emission signal following removal of the electric field was accompanied by a blue shift in fluorescence peak position (Figure S5). The PEF band shifts (λ_shift_ = −22 nm) from 572 to 550 nm. This was combined with changes in the fwhm (Figure S5). When the electric field was removed, the fluorescence peak FHWM narrowed from 60 to 25 nm as the PEF emission intensity increased. A similar effect was seen for the fluorophores RhB and TMPyP (Figures S3–S5) QDs (λ = 665 nm) (Figure S6). Turning off the electric field produced a spike in PEF with an ∼20-fold increase in PEF intensity for both molecular fluorophores (RhB and TMPyP) in comparison to the PEF signal recorded at 0 V (Figures S3 and S4).

The shift of the emission peaks of TMPyP in the presence of an electric field could be ascribed to the formation of a π–π complex between TMPyP and the template, in agreement with the report that π–π stacking interactions between, for example, nucleobases and TMPyP lead to shifts in emission as well as increased fluorescence intensity from TMPyP.^45^ Interestingly, the peak intensity at 655 nm was higher than that at 730 nm with increasing electric field (Figure S4); however, during charging and relaxation, the intensities of those bands were at the same level (Figure S4), meaning that changes in electronic structure due to a strong chemical interaction with the template could possibly occur simultaneously.^46,47^ Turning off the electric field produced a spike in PEF with an ∼20-fold increase in PEF intensity for both molecular fluorophores (RhB and TMPyP) in comparison to the PEF signal recorded at 0 V. A correlation between narrowing of the fluorescence fwhm and band shift of the fluorescence band and PEF signal intensity spike was also seen as for the QD nanocrystal fluorophore.

The origin of the spike in PEF intensity following the removal of the electric field may arise from charge trapping and detrapping processes occurring in the peptide semiconductor–plasmonic metal hybrid material. Studies of low-density polyethylene under an electric field showed that the space charge decayed over some hundreds of seconds into shallow and deep traps.^43^ In our system, removal of the electric field may produce trapped charges located at the FFNT–AgNP interface. These trapped interface state densities last over many hundreds of seconds, supporting a strengthened electromagnetic enhancement of PEF emission for over 600 s after removal of the electric field (Figure 2c and Figures S3–S5). The interactions between the externally applied electric field and intrinsic dipolar electric field on the FFNTs may be an alternative reason for the enhancement in PEF signal following removal of the electric field. FFNTs have been used in supercapacitors, due to the increased functional surface area they provide,^44,45^ and are capable of storing charge in the presence of an external electric field.^44−46^ The FFNTs between the gold electrodes in the sample might act as capacitors, with charge being generated when voltage is applied to the electrodes. When the field is switched off, the charge is freed and then can relocate to the analyte molecules via the AgNPs and result in an enhancement of the PEF signal. One study^47^ has shown that the surfaces of FFNTs contain numerous hydrophilic channels (diameters of 10 Å) that can allow transport of charges and increase mobility.^47,48^ These channels may store charge that is then released (e.g., over a period of hundreds of seconds); the charge moving to the AgNPs from the FFNTs increases the electromagnetic enhancement mechanism for PEF.

The origin of the PEF emission intensity increase from the QD nanocrystals following the removal of electric field potential may also center on the quantum confined Stark effect.^43^ It is known that, when an external electric field is applied to a QD nanocrystal, the electron states of the QD move to lower energies, in contrast to the hole states, which shift to higher energies. This shift in energy levels lowers the overlap integral, which reduces the recombination efficiency of the system.^49^ Removal of the electric field and consequently removal of the quantum confined Stark effect result in the PEF emission increasing as the overlap integral increases. This coupled with a capacitance and/or trapped charge assisted enhancement of the plasmonic electromagnetic field strengthens the PEF emission more strongly in comparison to that when the DC field is on.

Reproducibility studies were undertaken using different samples or different positions of the same samples, revealing a variation of around 15–17% (Figure S15). The application of an electric field to the microfabricated chip in the absence of any fluorophore (or PMMA) was also investigated to assess what contribution the FFNT makes to the electric field induced fluorescence signal (Figure S16). It was found that for a system in the absence of any fluorophore a fluorescence signal was generated with an applied electric field (Figure S16). The fluorescence signal occurring at ∼420 nm was outside of the spectral window of the fluorophores studied here. This fluorescence signal was relatively weak in comparison to the fluorescence signal observed when fluorophores were added to the microfabricated chip. PEF emission from QD nanocrystals with an applied electric field (Figure 2c) shows spectral features at ∼450–500 nm, which are not assigned to the QD nanocrystal but can be assigned to the fluorescence from the FFNTs.

**Figure 4:**
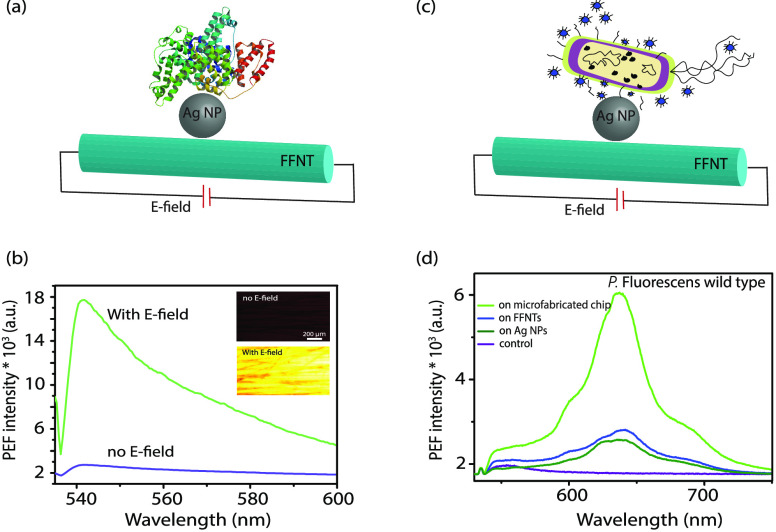
(a) Schematic drawing showing fluorescein-labeled bovine serum albumin (BSA) along with an AgNP-decorated FFNT. (b) PEF spectra (and images shown as an insert) recorded for the BSA-labeled protein (10^–8^ M) on the microfabricated chip with and without an applied electric field generated through the application of 20 V. (c) Schematic drawing showing *Pseudomonas fluorescens* (*P. fluorescens*) along with an AgNP-decorated FFNT. (d) PEF spectra of *P. fluorescens* wild type (10^7^ colony-forming units/mL) with and without an electric field.

We have also investigated the microfabricated chip device in the detection of fluorescently labeled proteins (Figure 4a,b and Figures S17–S19) to widen the use of our system in bioimaging applications. PEF measurements of BSA in the presence of an electric field show that there is around a 10-fold increase in PEF intensity in comparison with that for no field applied (Figure 4b and Figures S17–S19). This demonstrates a proof of principle that our microfabricated chip device with an applied electric field can potentially be used for small-protein detection and could further be used to investigate biomarkers for disease diagnostics.

The detection of bacterial pathogens such as *Pseudomonas fluorescens* (*P. fluorescens*) is of significant interest both in life science and in environmental research, due to the fact that microbial contamination can result in significant health concern.^50−55^ In this study, we have investigated the use of a microfabricated chip device in the detection of *P. fluorescens* and *Pseudomonas putida* (*P. putida*) strains (Figure 4c,d and Figures S20–S22). It can be clearly seen that using our microfabricated chip device with a voltage of 20 V results in an ∼11-fold increase (*P. fluorescens* mCherry), 6-fold increase (*P. fluorescens* wild type), and 10-fold increase (*P. putida* GFP) in PEF intensity in comparison to 0 V. The ability of the template to improve PEF intensity from different types of bacteria in water offers potential as a tool for early-stage detection of bacteria in water.

In conclusion, we have successfully demonstrated the enhancement of fluorescence intensity via a field-activated semiconductor–plasmonic nanostructure template from quantum dot nanocrystals and other dye molecules and bacteria. The plasmonic coupling between the fluorophores and the plasmonic–semiconductor microfabricated chip can be controlled by the electric field. The novel approach reported here can potentially improve high-efficiency light-emitting diode design and high-contrast imaging and provide a platform for biological and environmental monitoring. Given the excellent applicability of QDs for fluorescent biosensing, this approach can facilitate working at low molecule concentrations with maximum fluorescence enhancement and minimal background fluorescence.

## Materials and Methods

### Preparation of FFNT Solution

In order to prepare an FFNT solution, we dissolved the l-diphenylalanine peptide (Bachem, Bubendorf, Switzerland) in 1,1,1,3,3,3-hexafluoro-2-propanol (Sigma-Aldrich, Ireland) at an initial concentration of 100 mg/mL. The initial concentration of FFNTs was then further diluted in deionized water (ddH_2_O) to a final concentration of 2 mg/mL to allow the FFNTs to self-assemble. Fresh stock solutions were prepared for each experiment.

### Preparation of Microfabricated Chip

Si wafers (SiMat), cut to 2 cm × 1 cm, were cleaned by dipping in acetone for 2 min and then washed with ethanol and isopropanol (Sigma-Aldrich). The chip was then rinsed with deionized water and subsequently blown dry using nitrogen.^12,13,34,35^ In order to make the microfabricated chip, sputter-coating was used through a 3D printed mask with openings of ∼0.1 mm to form interdigitated gold electrode pairs. To create a patterned region, a mask with one opening of 1 mm was placed above the gold electrode for the FFNTs to align during the self-assembly process. The process to form aligned FFNT–AgNP templates follows a previously reported method.^12,13,34,35^ Briefly, patterned regions are formed by growing a silicon oxide layer on a Si surface via ultraviolet or ozone exposure through a 0.3 cm mask opening. The difference in wettability between exposed and unexposed regions results in the alignment of FFNTs during their self-assembly when a drop of FF solution with NPs is placed at the boundary between regions. The aligned FFNTs form in the hydrophilic regions due to the “repulsion” of the FF solution from the hydrophobic regions. Such chemical force gradients have been used to align other high-aspect-ratio materials.^12,13^

### Preparation of FFNT–AgNP Template

An AgNP solution at a concentration of 0.02 mg/mL in water was added to the heated FF (at 100 °C for approximately 2 min) and stirred for 3 min (1:1 ratio). The FFNT solution was heated at 100 °C to allow the FFNTs to disassemble and therefore ensure complete incorporation with metal NPs, as the FFNTs will reassemble again upon cooling to room temperature. Ag NPs will incorporate with FFNTs due to electrostatic interactions. Also, FFNTs have been reported to have the ability to bind with metal ions residing at a specific location that led to a red shift in absorption spectra. We have reported the effectiveness and working principle of these composites in our previous publications.^12,13,34,35,37^ Following this, 40 μL of the composite materials was then placed on the microfabricated chip to create the aligned FFNT–AgNP template on the SiO_2_ layer formed by UV/ozone. For the control sample, 60 μL of AgNPs (0.02 mg/mL) was drop-casted on a Si substrate.

### Probe Molecule Solutions

To prepare *meso*-tetrakis(*N*-methyl-4-pyridyl)porphine tetrachloride (TMPyP; T40125, Frontier Scientific) solutions, TMPyP powder was diluted with deionized water to a concentration of 10^–9^ M. Similarly, rhodamine B (RhB) (R6626-25G, Sigma-Aldrich, Ireland) and CdSe/ZnS alloyed quantum dots (1 mg/mL) (753866 and 753890, Sigma-Aldrich, Ireland) PMMA was prepared in toluene to a final concentration of 50 mg/mL. A mixture of TMPyP (or RhB) and PMMA was prepared by mixing both materials at a ratio of 1:5 for ∼10 min. Albumin-fluorescein isothiocyanate conjugate protein bovine (1002930814, Sigma-Aldrich, Ireland) was prepared with pH 7 Tris buffer solution (648315) and further diluted with deionized water to a concentration of 10^–6^ to 10^–8^ or 10^–9^ M. Crystal violet (CV) 1% aqueous solution (CAS No. 548-62-9) was diluted in distilled water to a concentration of 10^–8^ M.

### Spectral Characterization

Optical absorbance measurements (V-650, JASCO, Inc.) were performed with the microfabricated chip on a glass coverslip with electrodes during the application of voltage. The typical setup used is as follows: a 1 nm step size and bandwidth and a 400 nm/min scan speed. Spectra were recorded over a 190–900 nm wavelength range.

### Scanning Electron Microscopy (SEM)

A thin (∼8 nm) layer of gold was sputtered (Hummer IV, Anatech USA) on the samples before SEM imaging (JSM-7600F, JEOL).

### Fluorescence Spectroscopy

PEF measurements were undertaken using a bespoke Raman system that consisted of an inverted optical microscope (IX71, Olympus), a monochromatic laser (green laser, ThorLabs) with a beam splitter and long-pass filter (RazorEdge, Semrock), a spectrograph (SP-2300i, Princeton Instruments), and a CCD camera (IXON, Andor).^12,13^ To focus the laser (532 nm wavelength, 5 mW incident power), a 50× objective was used. PEF spectra were collected with an exposure time of 1 s. A 30 μL sample of the analyte molecule TMPyP, RhB, or QDs with and without PMMA at a concentration of 10^–9^ M was deposited (drop-casting) above the aligned FFNTs in the presence and absence of AgNPs. The average of typically 10 measurements is reported. PEF measurements were performed during an *in situ* applied electric field generated through the application of 0–60 V, in steps of 5 V; the voltage was applied using a PEW0028 DC power supply, following a process reported previously.^12,35^ Electrical cables or bonding wire was used to connect the microfabricated chip using silver paint, and then a DC voltage was applied during *in situ* Raman measurements as shown in Figure 1a. Relaxation was also recorded after removing the applied electric field or by applying low electric field values. The current flow in the microfabricated chip was measured using a TENMA digital multimeter (72-7725).

### Work Function Measurements

The work function of FFNTs was estimated on the basis of previously reported Kelvin probe force microscopy (KPFM) data.^34^ Briefly, the contact potential difference (CPD) (from KPFM measurements) is defined by
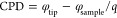
where φ_tip_ and φ_sample_ are the work functions of the tip and sample, respectively, and *q* is the elementary charge. The difference in CPD between SiO_2_ and FFNTs was reported to be ΔCPD ≈ 1.5 V.^34^ From the preceding equation and on the assumption that the work function of SiO_2_ is ∼4.7 eV, the estimated work function of the FFNTs is ∼6.2 eV.^34^

### Fluorescence Images

Fluorescence images were recorded using a fluorescence microscope (Zeiss AxioImager M1) with 20× and 50× objectives, an exposure time of 10 ms, and excitation wavelengths of 300, 500, and 600 nm.

### Organism and Culture

Four *Pseudomonas* strains were used to observe the effects of bacterial adhesion. These were *P. fluorescens* wild type, *P. fluorescens* mCherry, *P. putida* wild type and *P. putida* GFP. *P. putida* cultures were obtained by inoculating 50 mL of King’s B broth supplemented with tetracycline at a final concentration of 10 μg/mL using single colonies grown on King B agar at 28 °C. *P. fluorescens* cultures were obtained by inoculating 50 mL of LB broth supplemented with gentamicin at a final concentration of 10 μg/mL using single colonies grown on LB agar at 28 °C. The cultures were incubated at 28 °C with shaking at 75 rpm and left to grow to the mid-exponential stage, corresponding to optical densities of 2.2. Next, the culture was centrifuged at 5000 rpm for 10 min and the bacteria were added to 200 mL of 0.9% NaCl. For each organism, the final concentration was 10^7^ colony-forming units/mL. Next, 40 μL of this solution was drop-casted above the template and dried at 30 °C for 15–40 min.

### Theoretical Calculations

The full quasi-particle band structure of neutral Ag was calculated by starting from approximate Kohn–Sham density functional theory using SG15 optimized norm-conserving Vanderbilt scalar-relativistic pseudopotentials^56,57^ with the Perdew–Burke–Ernzerhof (PBE) exchange-correlation functional^58−60^ within the Quantum Espresso software.^61,62^ One-shot *G*_0_*W*_0_ simulations to obtain the approximate quasi-participle band structure and random-phase approximation^63−65^ simulations were performed using the Yambo software.^66^ For the charged Ag cases, the quasi-particle band structure was assumed to be fixed, while the Fermi levels were shifted for each given charge. Furthermore, the Drude plasmon parameters for each nominal charge were obtained using in-house code combined with the semiempirical protocol for the inverse lifetimes introduced in ref (44). The localized-surface plasmon population spectra were obtained by the product of the theoretical EELS and the quality factor discussed in details in ref (44) and its references.

## Supporting Information Available

SEM, fluorescence, photoluminescence, and UV–vis data and theoretical calculations (PDF)
