# Supplementary material for: Electric Field Tunability of Photoluminescence from a Hybrid Peptide–Plasmonic Metal Microfabricated Chip
Source: JACS Au. 2021 Oct 8;1(11):1987–95. doi: 10.1021/jacsau.1c00323 (PMC8611722; doi:10.1021/jacsau.1c00323)
Supplement: Supplementary file 1 — au1c00323_si_001.pdf [file au1c00323_si_001.pdf]

Electric field tunability of photoluminescence from a hybrid peptide-plasmonic metal microfabricated chip

Sawsan Almohammed [a, b], Okan K. Orhan [c], Sorcha Daly [d], David D. O'Regan [c], Brian J. Rodriguez * [a,b], Eoin Casey [d], and James H. Rice * [a]

[a]School of Physics, University College Dublin, Belfield, Dublin 4, Ireland
[b]Conway Institute of Biomolecular and Biomedical Research, University College Dublin, Belfield, Dublin, Ireland, D04 V1W8

[c]School of Physics, AMBER, and CRANN Institute, Trinity College Dublin, the University of Dublin, Dublin, Ireland, D02 PN40

[d]School of Chemical and Bioprocess Engineering, University College Dublin, Belfield, Dublin, Ireland D04 V1W8

Supplementary information

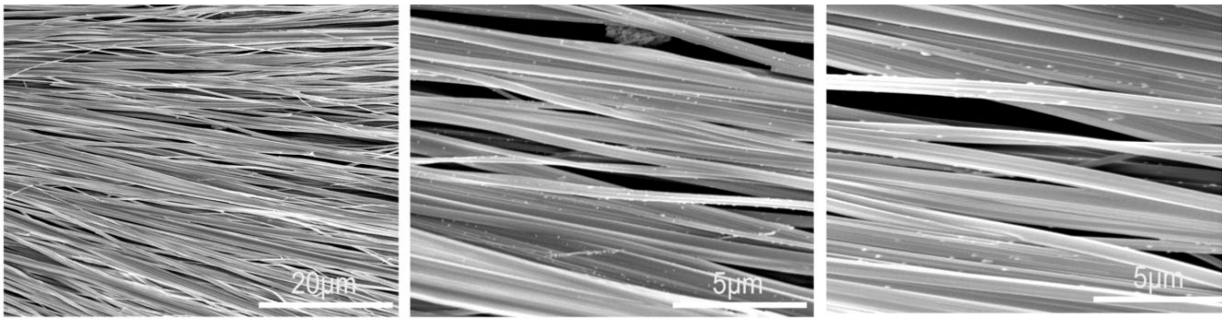

Fig. S1. (a, b) SEM images of aligned diphenylalanine nanotube Ag nanoparticle (FFNT–AgNP) template and after the addition a fluorophore molecule, indicating the stability of the template.

A microfabricated chip design based on aligned FFNTs and AgNPs were prepared on Si substrates (shown schematically in Fig. 1(a)), following a process reported previously.[1,2] Briefly, gold electrodes were sputtered through openings in a 3D printed mask on Si substrates. Wettability-patterned regions were generated by growing a silicon oxide layer (contact angle of 4 ± 1°) on a Si surface (contact angle of 69 ± 2°) via selective UV/ozone exposure. Following that, a heated solution of FFNTs and AgNPs (100 °C for 5 minutes) was deposited in the hydrophilic region. Upon drying at room temperature, the difference in wettability between exposed and unexposed regions led to the alignment of the AgNP-decorated FFNTs during the self-assembly process. The average distance between NPs is 30 ± 22 nm (n = 200 NPs) with a density of 152 ± 43 NPs/μm^2, as determined from SEM images using ImageJ.

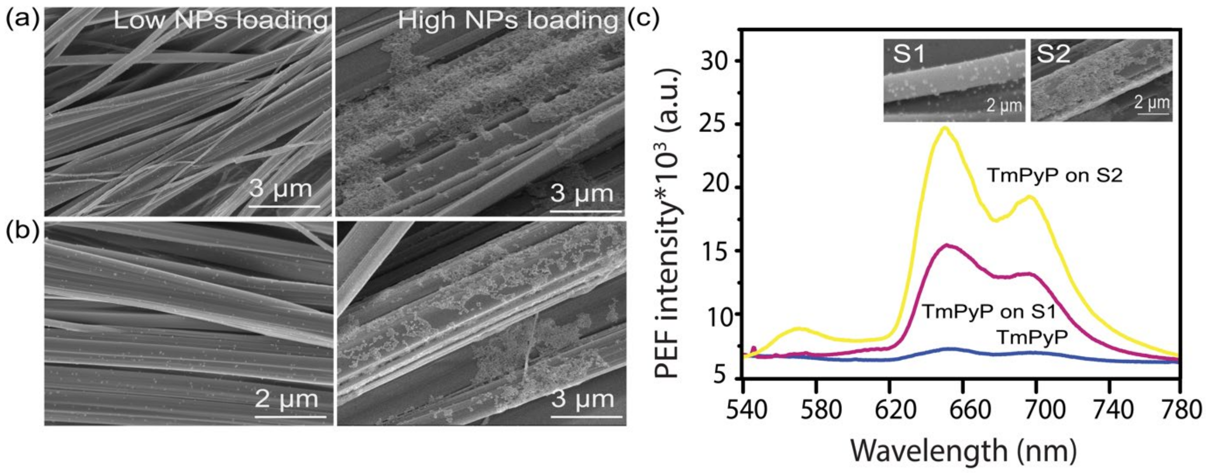

Fig. S2. (a, b) SEM images of aligned FFNT–AgNP templates prepared low and high NP loading. (c) PEF spectra recorded for TMPyP on the microfabricated chip. S1 was prepared at low NP loading (FFNT:AgNP ratio ~1:0.5) and S2 at high NP loading (~1:2) .

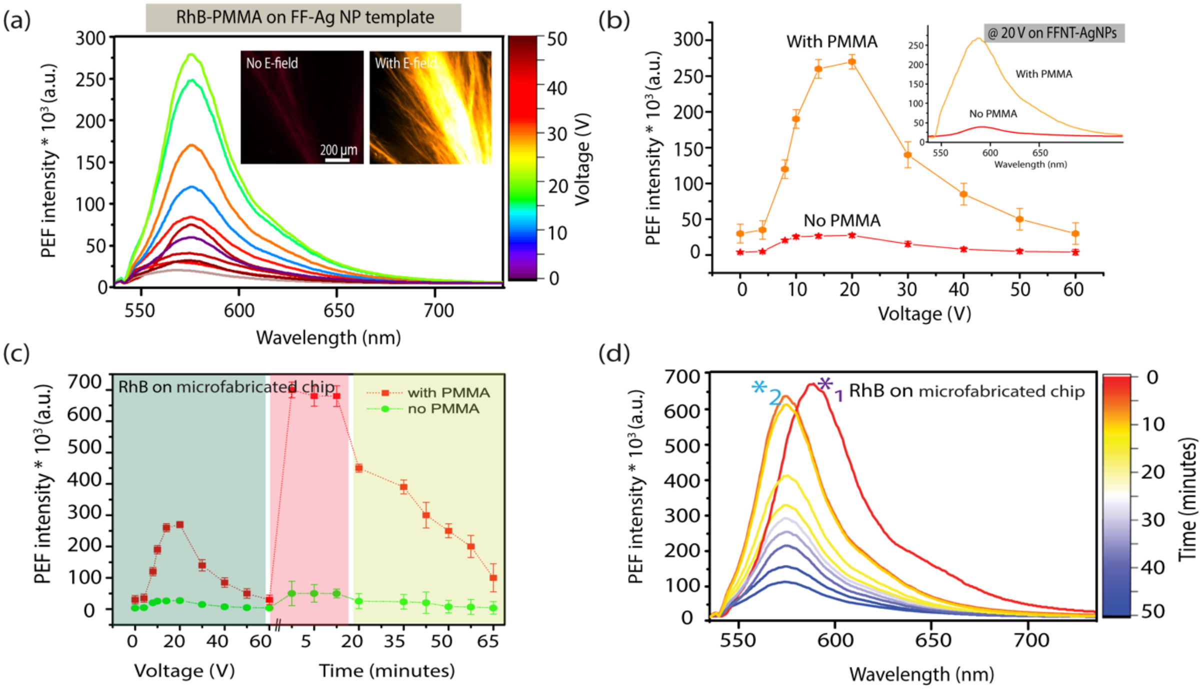

Fig. S3. PEF spectra recorded for RhB. **(a)** PEF spectra recorded for RhB with and without an applied voltage (0–50 V). The inset in (a) is fluorescence image excited at 500 nm, without and with E-field. **(b)** Plot of PEF signal intensity with and without a dielectric polymer matrix (PMMA), in which the fluorophore was embedded, for applied voltages of 0–60 V. The inset in (b) shows PEF spectra recorded for RhB with and without PMMA in the presence of an electric field generated through the application of 20 V. **(c)** Plot of PEF emission spectra intensity as a function of applied voltage and with the removal of electric field as a function of time following removal of electric field. **(d)** PEF emission measurements from RhB recorded after the removal of electric field.

Studies have shown that the addition of a thin polymer layer can introduce a ~nanometer-thick distance between the analyte and the substrate, which can prevent quenching.[3,4] For this reason, fluorophores at a concentration of ~10^{-9} M were mixed with a dielectric polymer (PMMA) prior to PEF measurements, and then deposited above the microfabricated chip device. The use of PMMA enables control over the competition between enhancing and quenching processes.

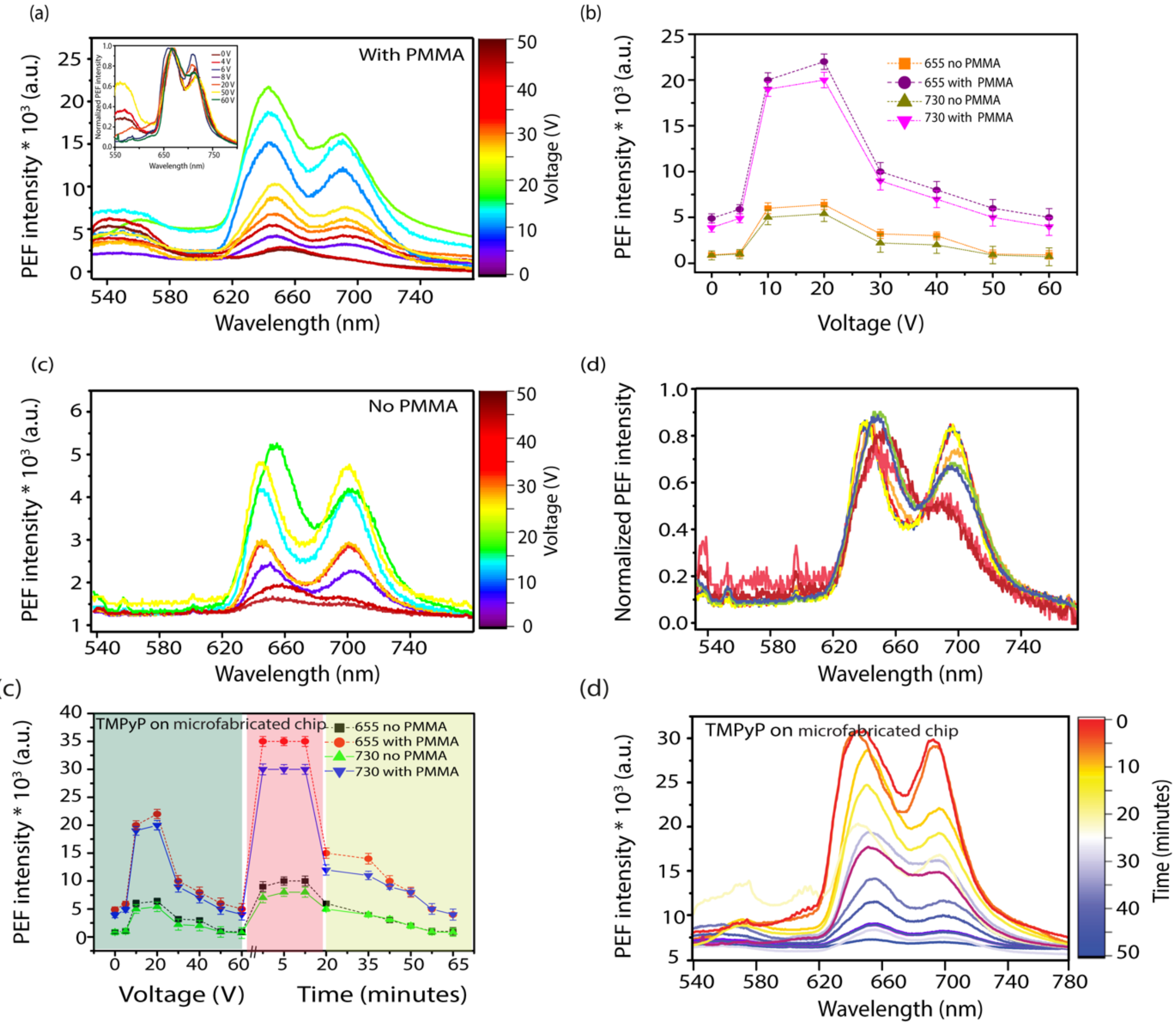

Fig. S4. PEF spectra recorded for TMPyP. **(a)** PEF spectra recorded for TMPyP with and without an applied electric field generated through the application of 0–50 V. Inset shows the normalized spectra shown in (a). **(b)** Plot of PEF signal intensity with applied electric field generated through the application of 0–60 V with and without the use of PMMA. **(c)** PEF spectra recorded for TMPyP with and without an applied electric field generated through the application of 0–50 V in the absence of PMMA. **(d)** Normalized data presented in (c). **(e)** Plot of PEF emission spectra intensity as a function of applied voltage and with the removal of electric field as a function of time following removal of electric field. **(f)** PEF emission measurements from TMPyP recorded after the removal of electric field.

Interestingly, the peak intensity at 655 nm was higher than that at 730 nm with increasing electric field (Fig. S4), however, during charging and relaxation, the intensity of those bands became the same level (Fig. S4), meaning that changes in electronic structure due to strong chemical interaction with the template could possibly occur simultaneously.[5,6] It should be noted that the shift of the emission peaks of TMPyP in the presence of electric field could be ascribed to the formation of a π-π complex between TMPyP and the template, in agreement with the report that π-π stacking interactions between, e.g., nucleobases and TMPyP lead to shifts in emission as well as increased fluorescence intensity from TMPyP.[7–47]

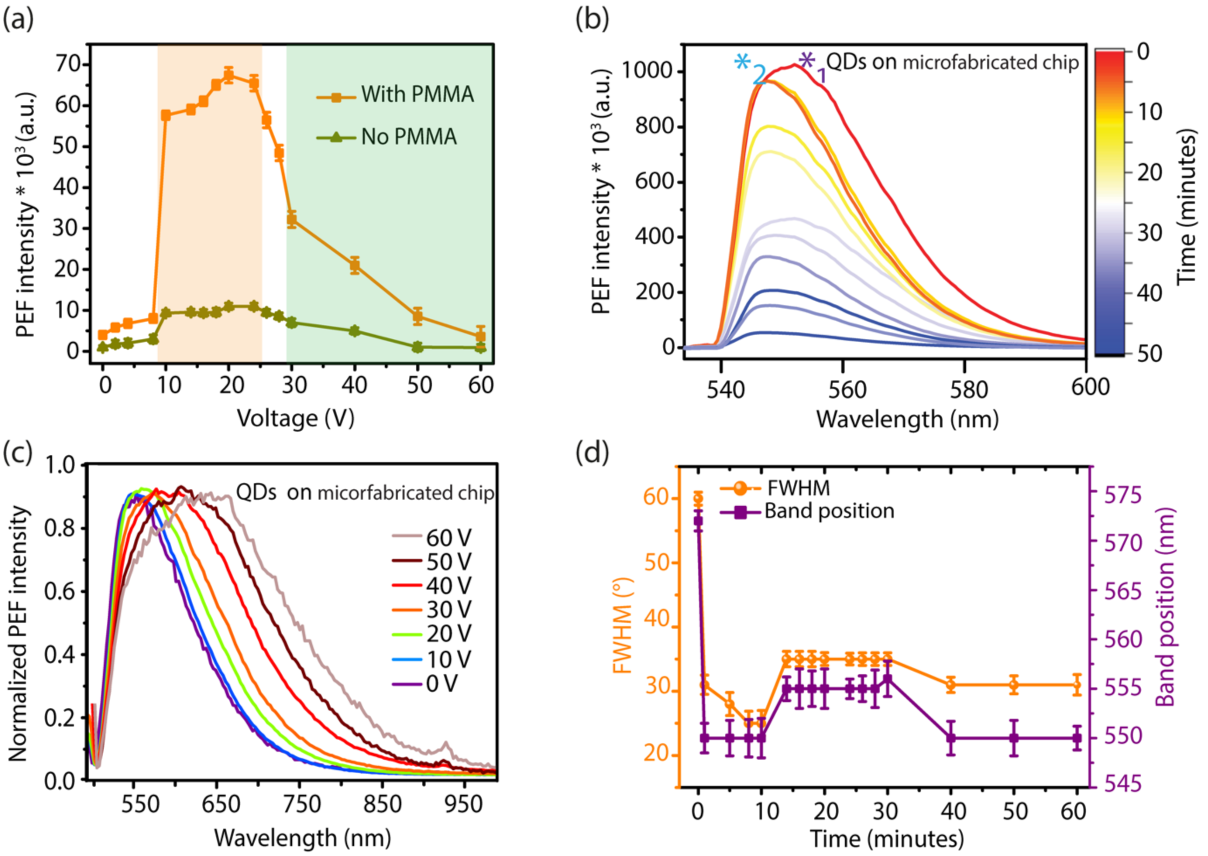

Fig. S5. PEF spectra recorded for QD nanocrystals. **(a)** PEF emission spectra recorded for QD nanocrystal fluorophores with an applied electric field from 0 to 60 V. and without an applied electric field. **(b)** PEF spectra recorded for the QD nanocrystal fluorophore with variable applied electric field generated through the application of 0–60 V.**(c)** Normalized spectra recorded for the QD nanocrystal fluorophore with variable applied electric field generated through the application of 0–60 V. **(d)** Plot of PEF emission spectra band position and FWHM, as a function of time following removal of electric field.

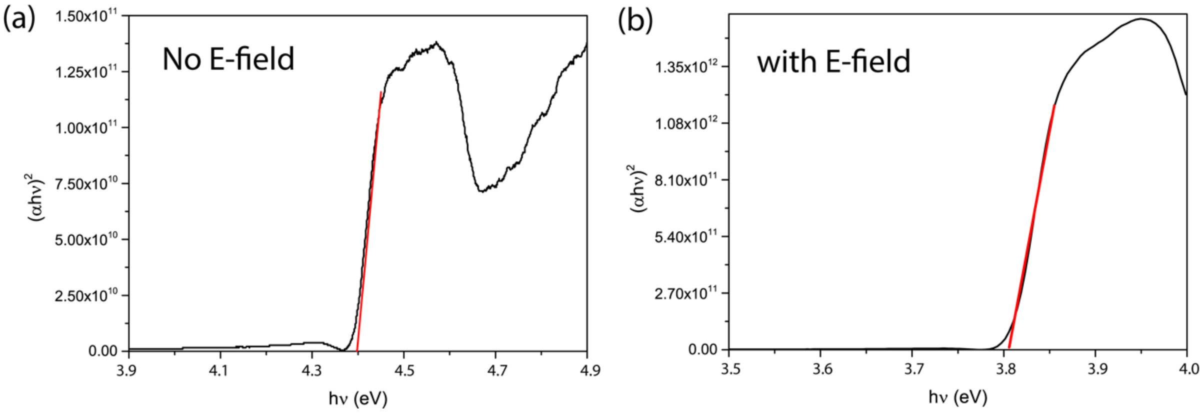

Fig. S6. Band gap determination from UV-vis measurements of FFNTs with and without E-field shown reduction of the band gap with the application of electric field. This data showing that in the absence of an electric field, a FFNT is a semiconducting material with band gap of ~4.4 eV; however, in the presence of an electric field, the band gap can be reduced to ~3.8 eV, meaning that a FFNT becomes easier to be excited and strongly coupled with NPs enabling effective charge transfer from the nanotube to the metal nanoparticles.

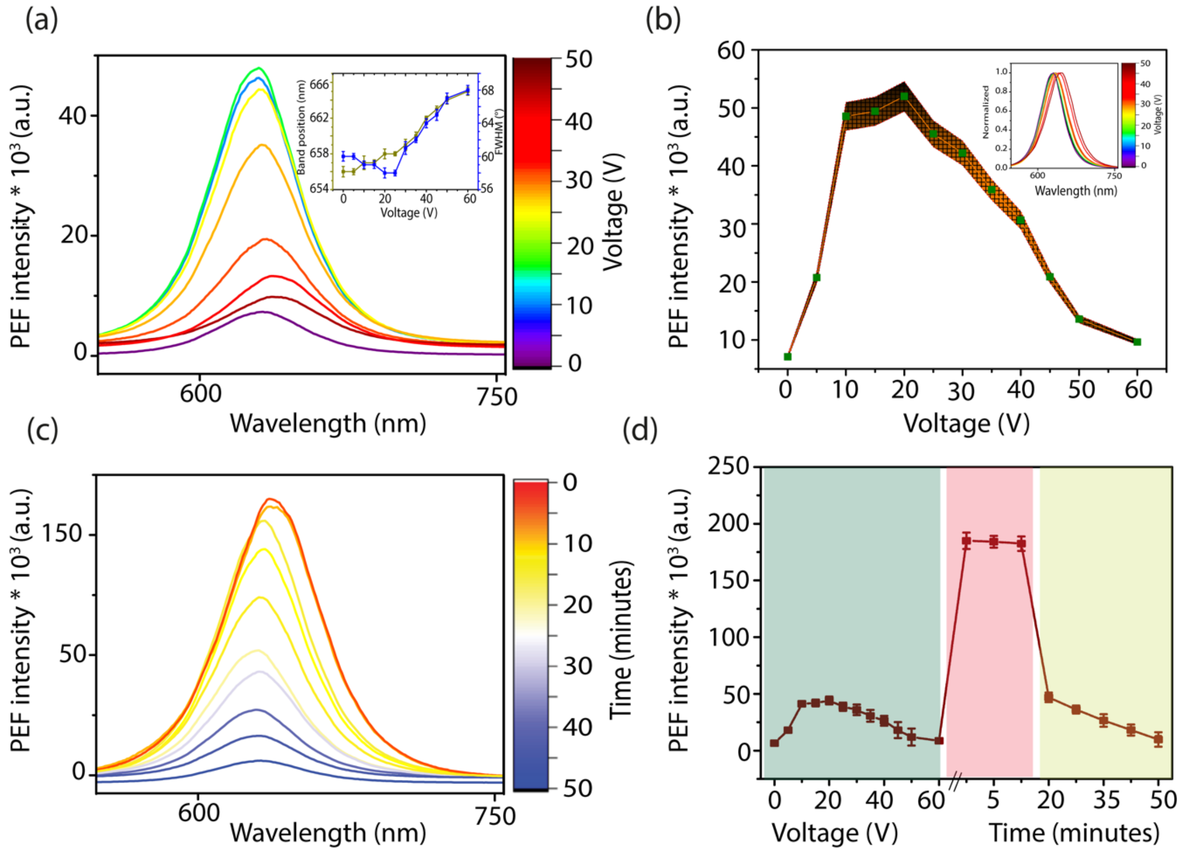

Fig. S7. (a) PEF spectra recorded for QDs (λ = 665 nm) and PMMA on the microfabricated chip with and without an applied electric field generated by applying 0–50 V. The inset in (a) shows PEF emission spectra band position and full width half maximum (FWHM) with applied voltage. (b) PEF signal intensity with applied voltage. The inset in (b) is normalized data presented in (a). (c) PEF emission spectra recorded over time after the removal of the electric field. (d) PEF emission spectra intensity as a function of voltage and as a function of time following the removal of the electric field.

For the Purcell effect to be present there is a requirement for accelerating the radiative recombination (the Purcell effect) through a resonant overlap of the plasmon frequency with the PL band of the emitter. Data from the paper potentially supports this possibility. As outlined in page 6 we report that for instance, QDs with λ = 525 nm yield higher enhancement than that QDs with λ = 665 nm; a ~14-fold increase and a ~8-fold increase, respectively, as we use a laser excitation wavelength of 532 nm (Fig. S7). Such an effect may arise from a Purcell effect. However, with the application of electric field, the SPR of the metal can be tuned to be close to the energy of the excitation laser to provide additional coupling with the probe molecules under study. According to literature, the luminescence intensity could be further resonance-enhanced when the resonance peaks of metal nanoparticles are matched with the luminescence peaks of QDs. However, we now have added that the Purcell effect is a potential possibility in regard to the mechanism in addition to a strengthened electromagnetic field effect, such a combination of effects been reported in papers such as for example in.[9]

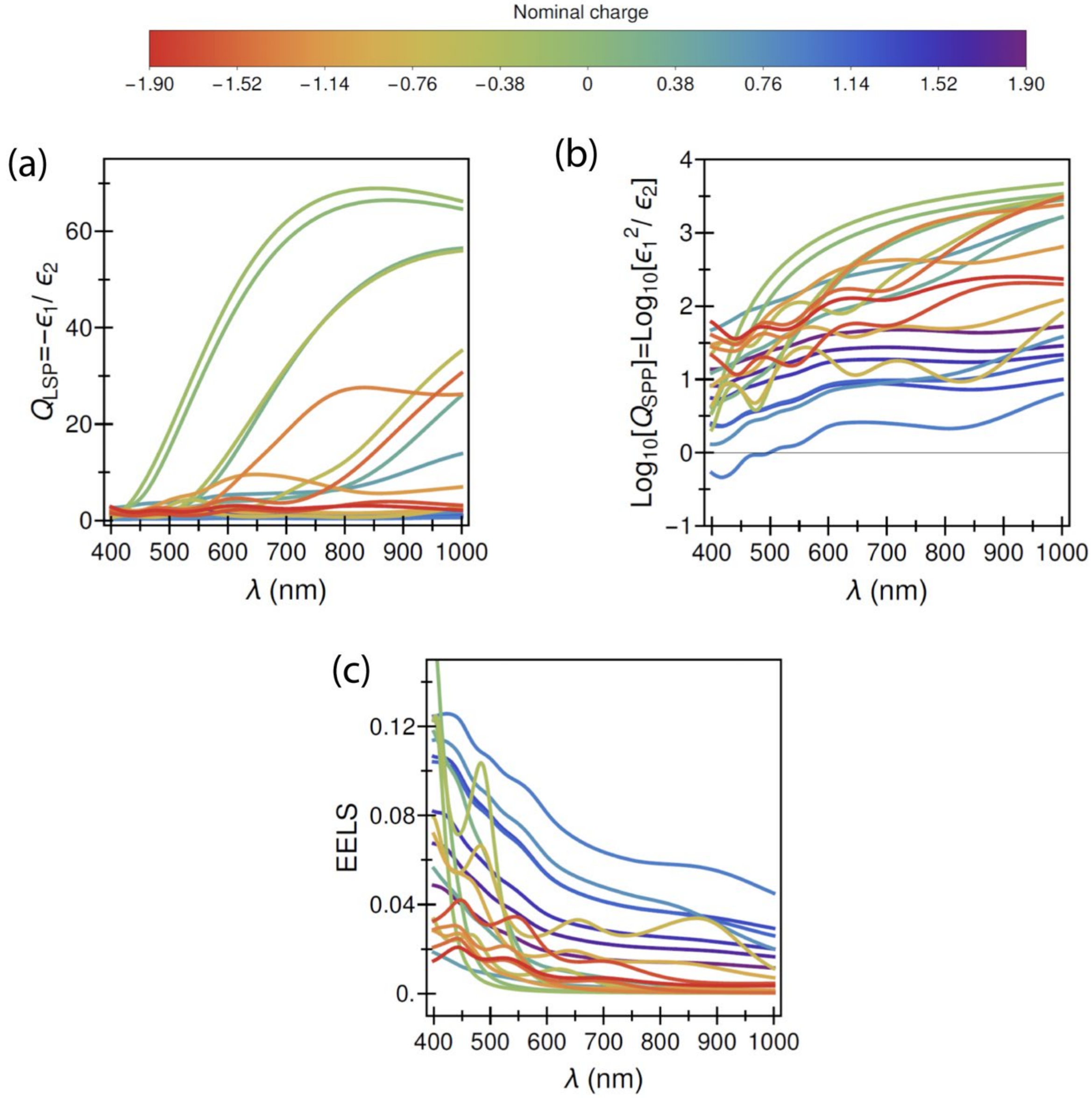

Fig. S8. Theoretical spectroscopy of bulk Ag charged positively and negatively (in units of the fundamental charge *e*). (a-b) Linear and log plots for the quality factor of the localized-surface plasmon (Q_{LSP}) as a function of wavelength. (c) EELS as a function of wavelength.

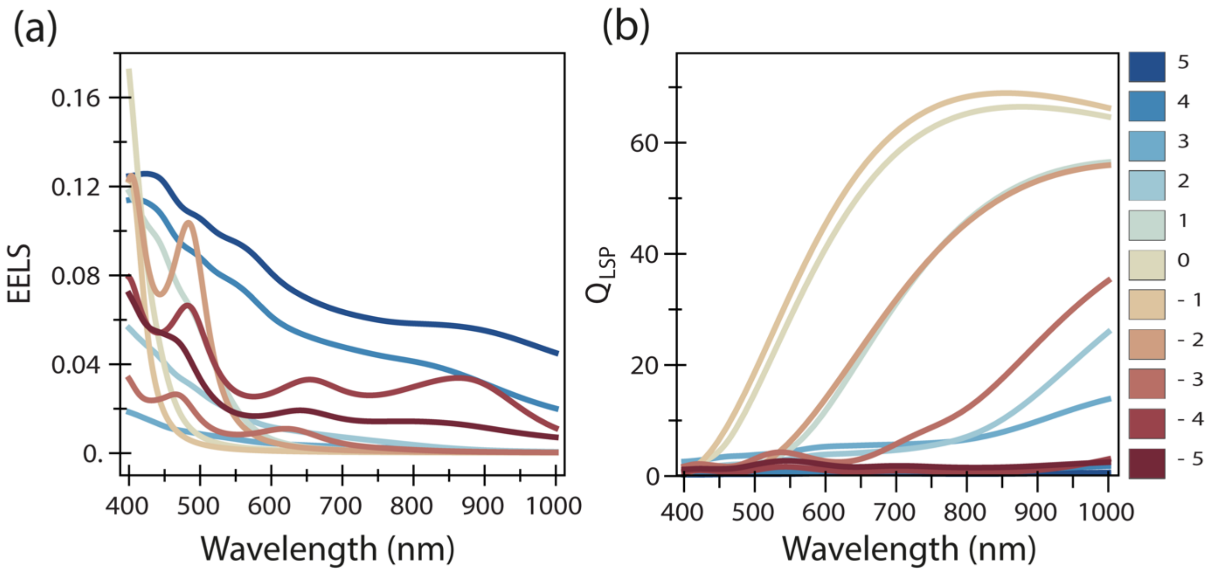

Fig. S9. Theoretical spectroscopy of the Ag charged by -%5 to %5 of the valence electron number of its neutral configuration (19 electrons), shown with a Gaussian smearing of 0.1 eV **(a)** Electron-energy loss spectroscopy. **(b)** Quality factor of the localized-surface plasmon.

The neutral Ag exhibits a strong localized-surface plasmon population (P_{LSp}) at ~410 nm, shown in Fig. 3 main paper (c), in close agreement with Fig. 1 main paper (c). However, this strong plasmonic peak generated mostly due to dense presence of the nearly free electron, hinted in the EELS in Fig. S9(a), is not very long-living at the given wavelength and expected to decay into inter-band transitions as its quality factor is quite small in Fig. 3 main paper (c). Negatively charging the bulk Ag reduced the intensity of plasmonic response; however, it shifts the primary peak by ~10 nm, and in the case of %3 of 19 valence electron negative charge, it generates a secondary peak ~500 nm in the EELS leading a more dominant secondary plasmonic generation at ~510 nm in Fig. 3 main paper (c). Hence, while neutral bulk Ag generated denser localized-surface plasmon at lower wavelengths, the negatively charged Ag may produce higher populated plasmon above 500 nm.

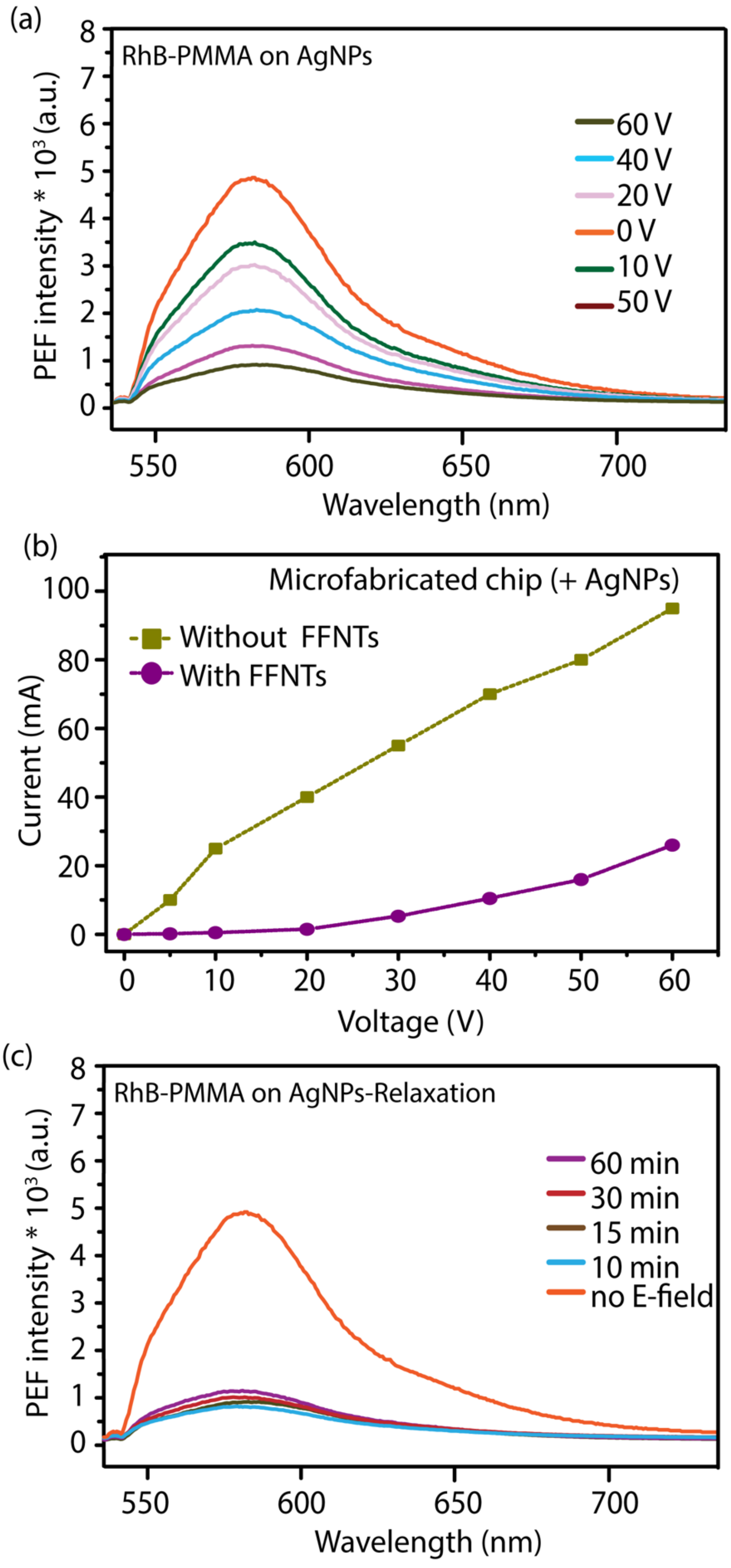

Fig. S10. (a) PEF spectra recorded for RhB with and without an applied electric field (E-field) generated through application of 0–60 V in the absence of FFNTs. (b) Plot of the measured current in the presence and absence of FFNTs as a function of the voltage applied (the current was measure using a multi meter). (c) Relaxation after turning off the electric field.

Having AgNPs only, in the absence of FFNTs, did not result in an increase in PFE intensity. On the contrary, significant and continuous reduction in the PEF signal was observed with applied electric field. This could be due to the occurrence of higher currents (Fig. S10).[8]

To verify this, we measured the current in the substrate during the application of electric field to be 118 mA at 60 V when only NPs were present. However, at the same voltage in the presence of FFNTs, a 26-mA current was measured. We have noticed that high currents lead to increased temperature. Metal NPs are easily oxidized in air resulting in loss of both signal activity as well as signal reproducibility.[8]

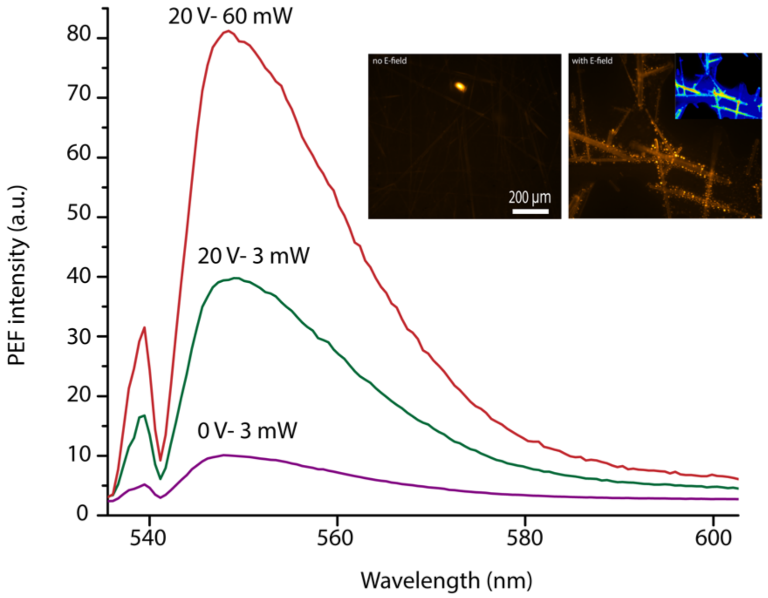

Fig. S11. PEF spectra recorded at different laser powers from QDs with PMMA on the microfabricated chip in the presence of an electric field generated through the application of 20 V and fluorescence images of the microfabricated chip with QDs with and without E-field.

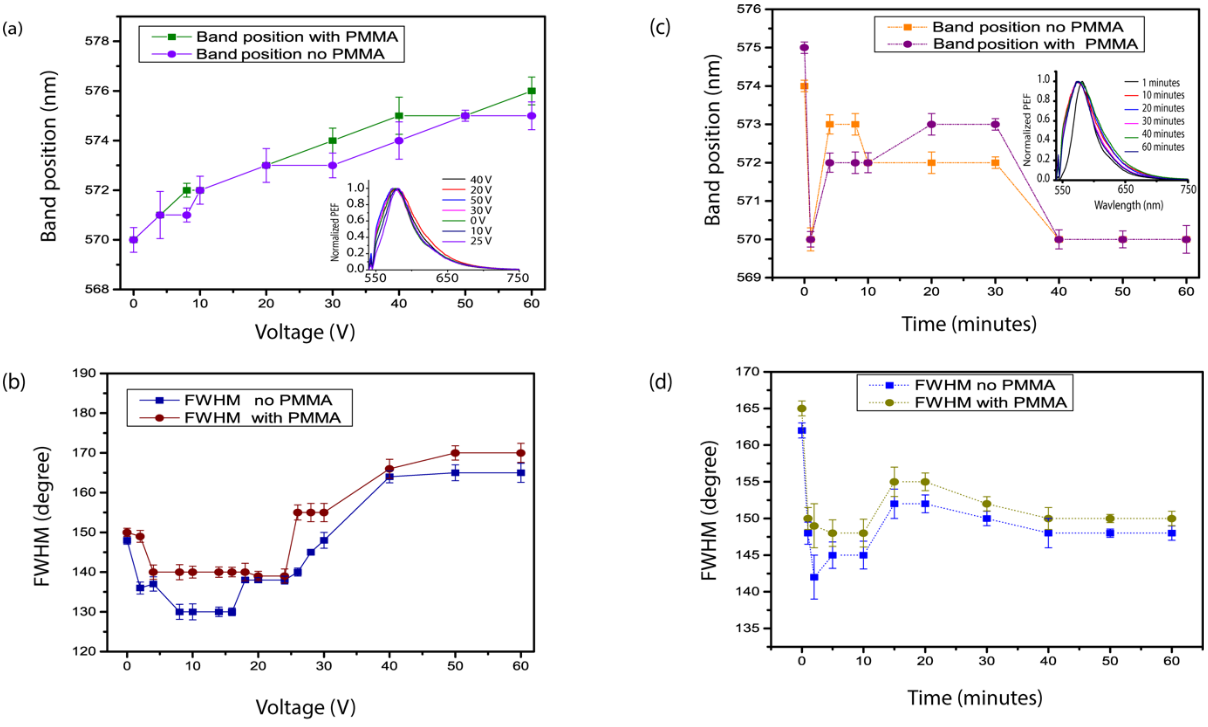

Fig. S12. PEF measurements from RdB in a PMMA matrix on the FFNT–AgNP template for applied voltages 0–60 V. (a,b) Effect of applied electric field on the PEF emission spectra. Plot of band position and FWHM from PEF emission spectra recorded as a function of applied voltage. (c,d) Effect of removal of the electric field. Plot of band position and FWHM from PEF emission spectra recorded as a function of applied voltage.

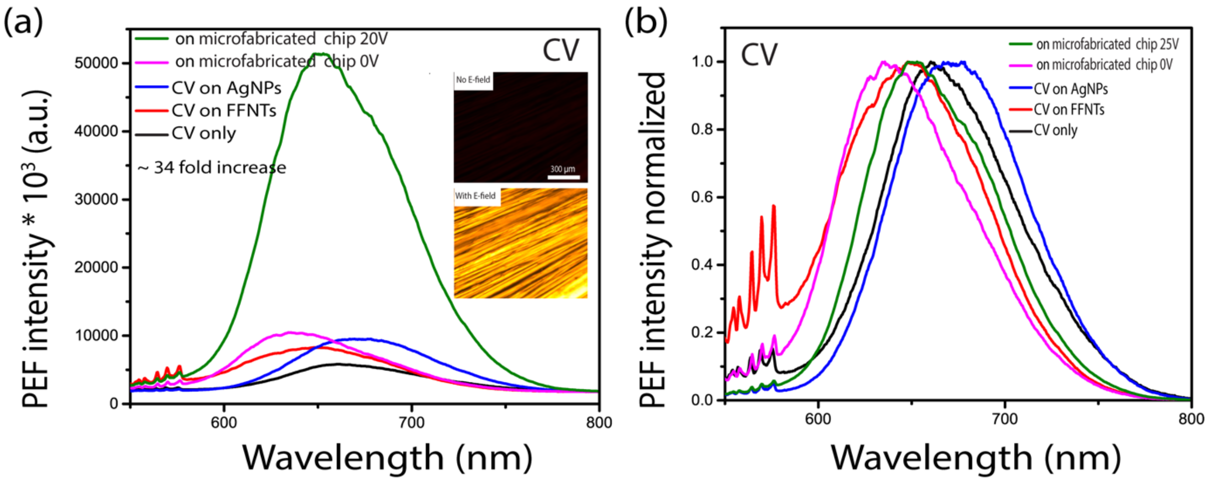

Fig. S13. (a) PEF spectra recorded for the CV (10^{-8} M) on the microfabricated chip with and without an applied electric field of 20 V, and images shown as an insert are fluorescence images of the microfabricated chip with CV without (up) and with electric Field down), (b) is normalized data presented in (a).

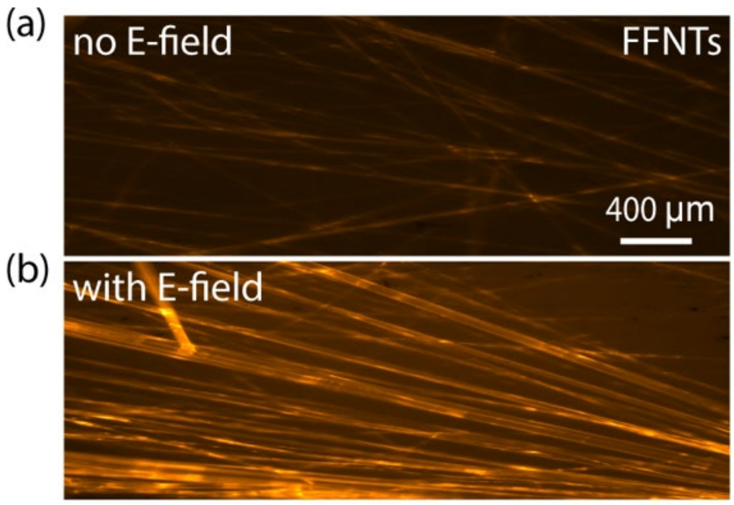

Fig. S14. Fluorescence images of FFNTs without (a) and with (b) E-field.

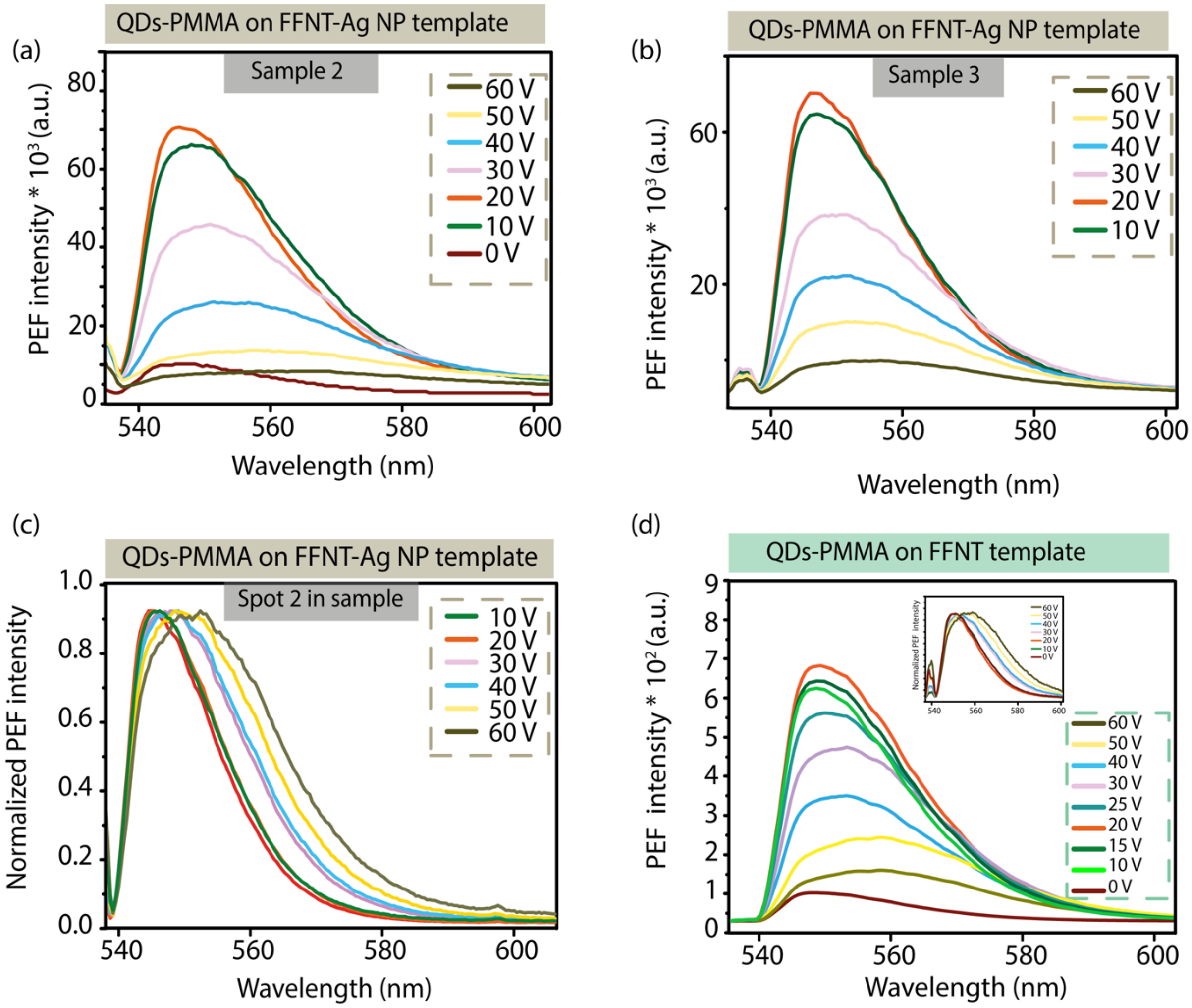

Fig. S15. (a-c) PEF spectra recorded for QDs with and without an applied electric field generated through application of 0–60 V. (d) PEF spectra recorded for QDs with and without an applied electric field generated through application of 0–60 V on the microfabricated chip without AgNPs. Inset shows the normalized spectra shown in (d).

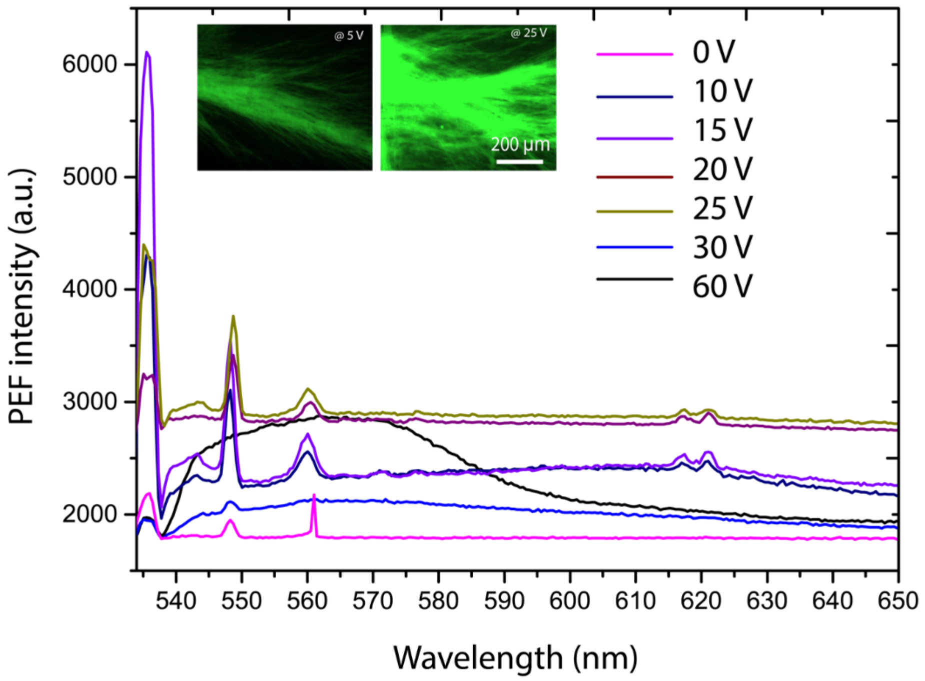

Fig. S16. PEF measurements from the FFNT template for applied voltages of 0–50 V and fluorescence images of the microfabricated chip with and without E-field.

The fluorescence signal occurring at ~420 nm was outside of the spectral window of the fluorophores studied here. This fluorescence signal was relatively weak in comparison to the fluorescence signal observed when fluorophores were added to the microfabricated chip. PEF emission from QD nanocrystals with an applied electric field (Fig. 2(c)) shows spectral features at ~450–500 nm, which are not assigned to the QD nanocrystal, but can be assigned to the fluorescence from the FFNTs.

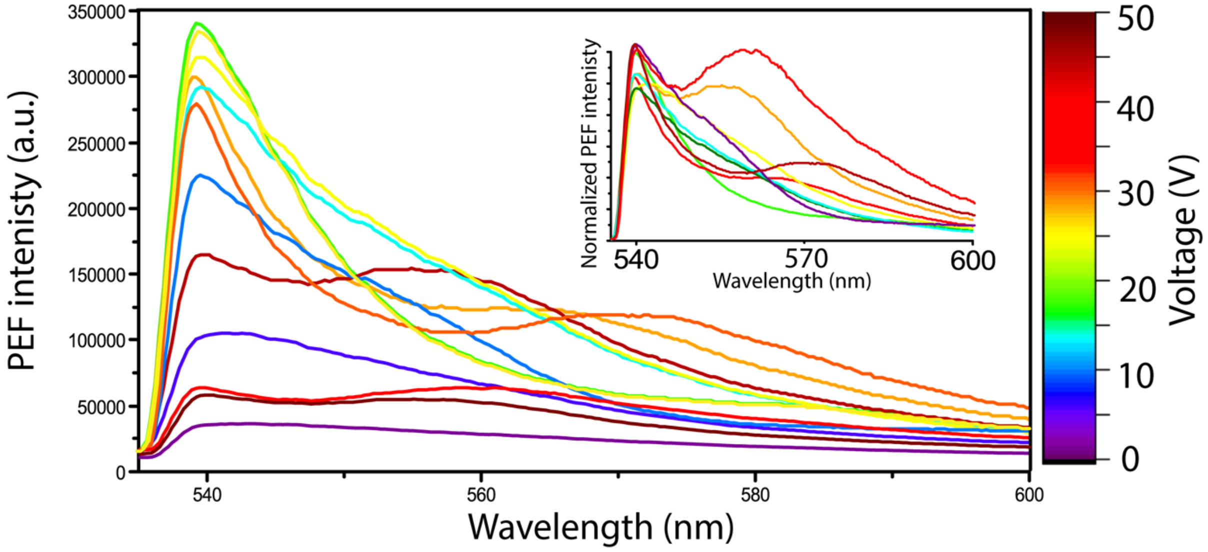

Fig. S17. PEF measurements from BSA with the FFNT–AgNP template for applied voltages 0–50 V; the normalized spectra are shown in the inset.

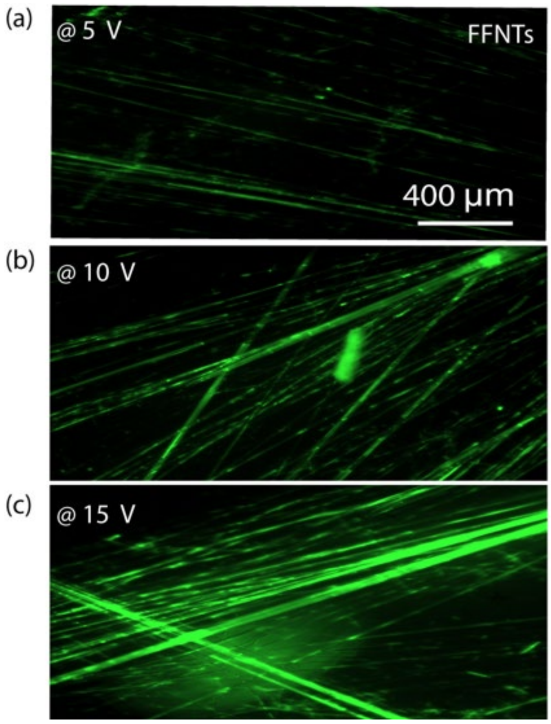

Fig. S18. Fluorescence images of FFNTs with probe molecule BSA with E-field at different DC voltages As the voltage increases, fluorescence intensity also increases.

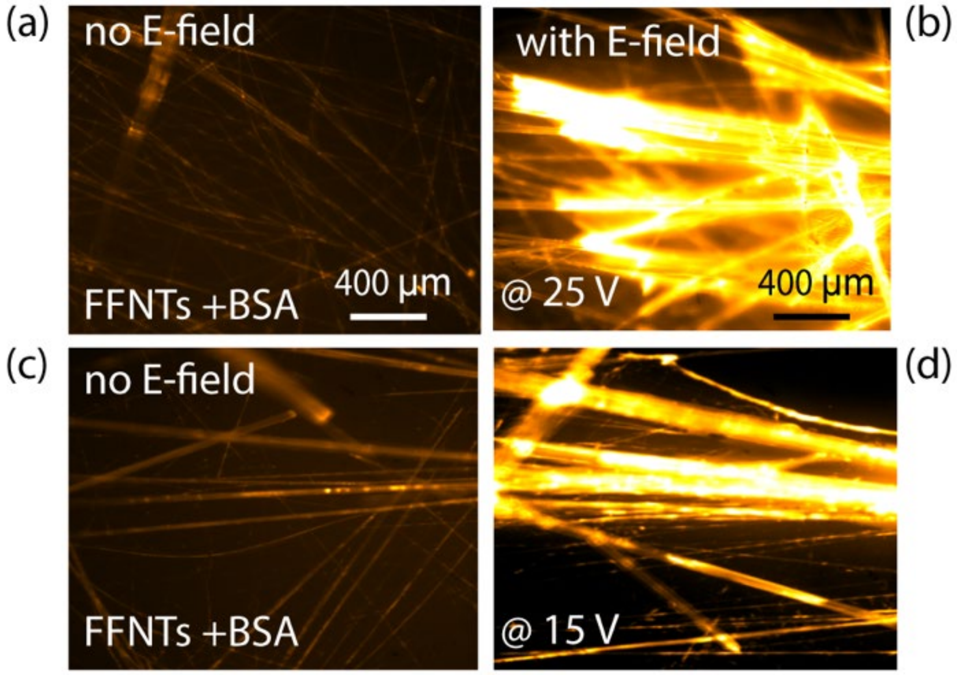

Fig. S19. Fluorescence images of FFNT–AgNP template with probe molecule BSA without (a-c) and with (b-d) E-field at different voltages.

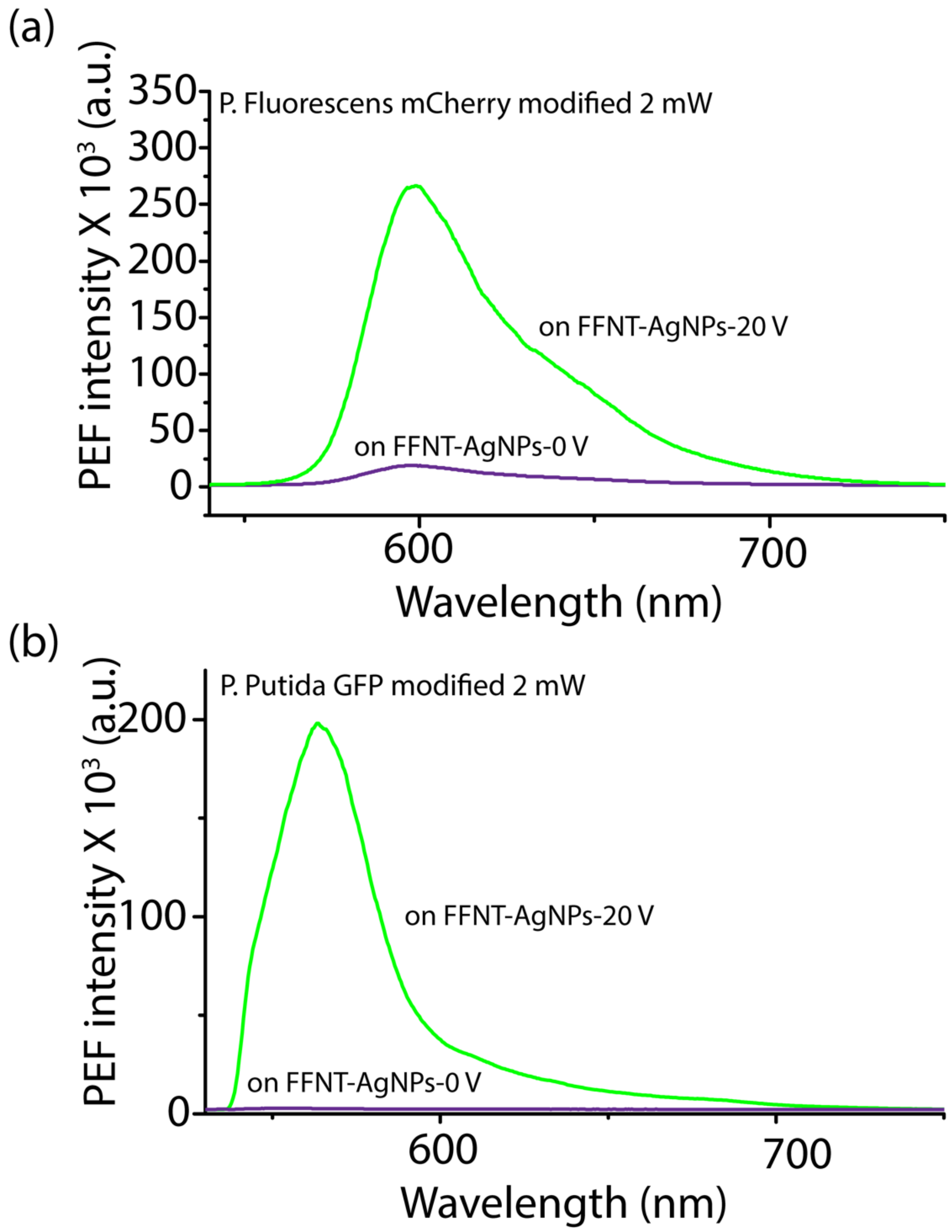

Fig. S20. PEF spectra of *Pseudomonas fluorescens* (*P. fluorescens*) mCherry (10^7 CFU/ml) (a) and *Pseudomonas putida* (*P. Putida*) GFP (10^7 CFU/ml) (b) on the FFNT–AgNP microfabricated chip with and without E-field.

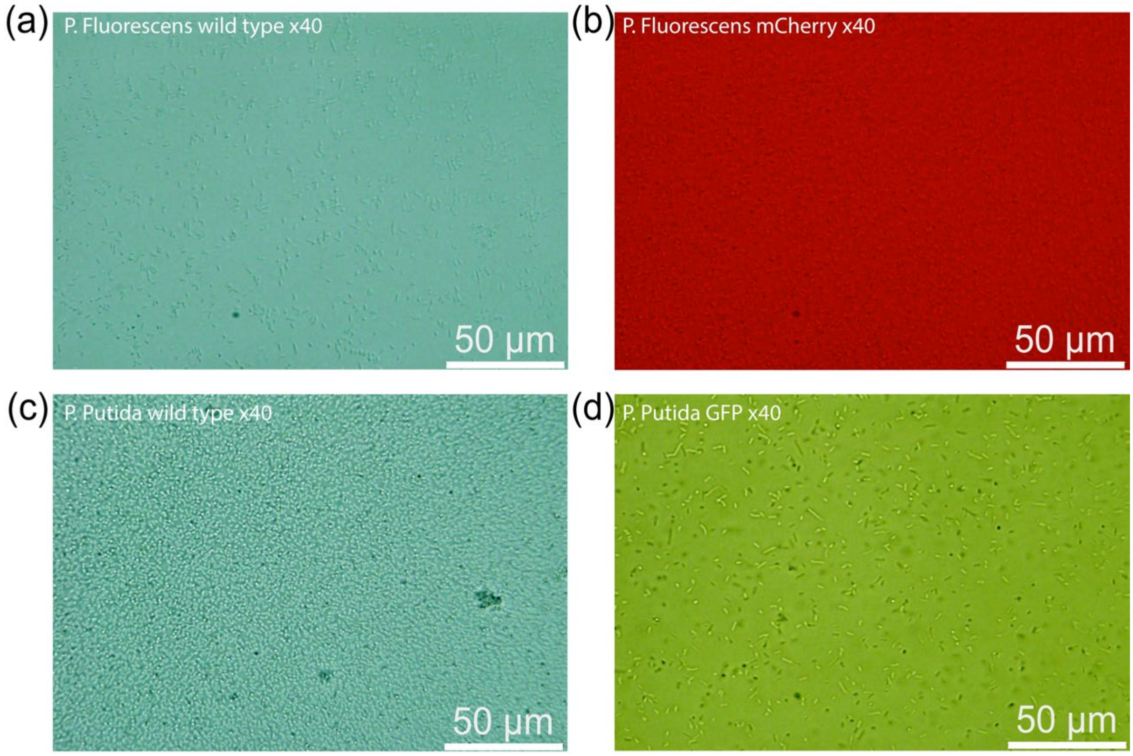

Fig. S21. Fluorescence microscopy was used to observe the organisms *P. fluorescens* wild type (a), *P. fluorescens* mCherry (b), *P. putida* wild type (c) and *P. putida* GFP (d). An Olympus BX51 microscope was used to image the cells suspended in a 0.9% NaCl solution in 6 well plates. Images were taken at x40 magnification.

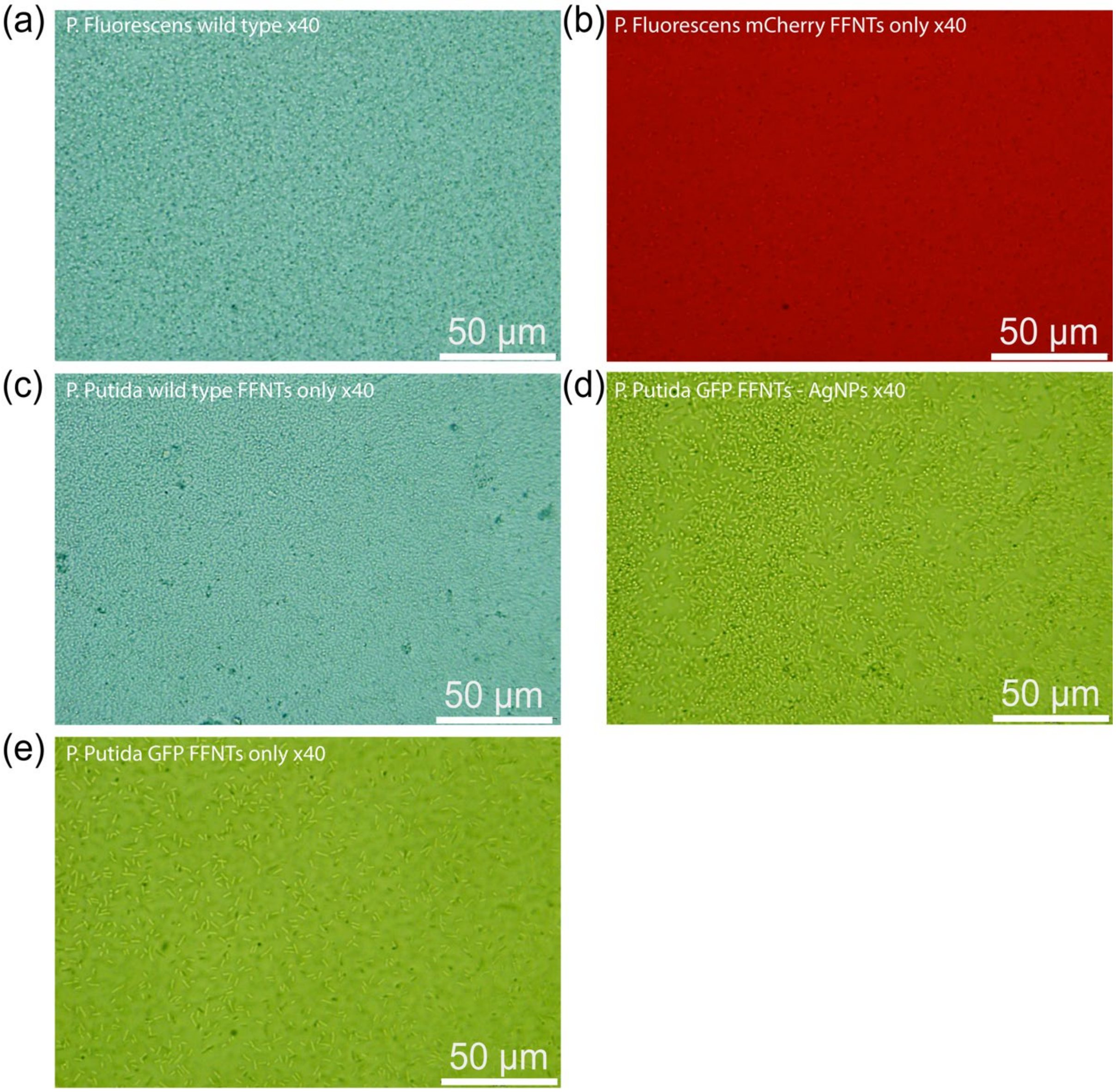

Fig. 22. Fluorescence microscopy was used to observe *P. fluorescens* wild type with FFNTs (a), *P. fluorescens* mCherry with FFNTs (b), *P. putida* wild type with FFNTs (c) *P. putida* GFP with FFNTs and AgNPs (d) and *P. putida* GFP with FFNTs only. An Olympus BX51 microscope was used to image the cells suspended in a 0.9% NaCl solution in 6 well plates. Images were taken at x40 magnification.

References

(1) Almohammed, S.; Zhang, F.; Rodriguez, B. J.; Rice, J. H. Electric Field-Induced Chemical SERS Enhancement from Aligned Peptide Nanotube–Graphene Oxide Templates for Universal Trace Detection of Biomolecules. *J. Phys. Chem. Lett.* **2019**, 10 (8), 1878-1887.

(2) Almohammed, S.; Tade Barwich, S.; Mitchell, A. K.; Rodriguez, B. J.; Rice, J. H. Enhanced Photocatalysis and Biomolecular Sensing with Field-Activated Nanotube-Nanoparticle Templates. *Nat. Commun.* **2019**, *10* (1), 2496. https://doi.org/10.1038/s41467-019-10393-9.

(3) Sun, J.; Li, Z.; Sun, Y.; Zhong, L.; Huang, J.; Zhang, J.; Liang, Z.; Chen, J.; Jiang, L. Uniform and Reproducible Plasmon-Enhanced Fluorescence Substrate Based on PMMA-Coated, Large-Area Au@Ag Nanorod Arrays. *Nano Res.* **2018**, *11* (2), 953–965. https://doi.org/10.1007/s12274-017-1708-y.

(4) Jie, Y.; Yonghua, L.; Pei, W.; Hai, M. Integral Fluorescence Enhancement by Silver Nanoparticles Controlled via PMMA Matrix. *Opt. Commun.* **2011**, *284* (1), 494–497. https://doi.org/10.1016/j.optcom.2010.09.007.

(5) Habib, M. A.; Sarker, A. K.; Tabata, M. Interactions of DNA with H2TMPyP4+and Ru(II)TMPyP4+: Probable Lead Compounds for African Sleeping Sickness. *Bangladesh Pharm. J.* **2015**, *17* (1), 79–85. https://doi.org/10.3329/bpj.v17i1.22321.

(6) Ishida, Y.; Shimada, T.; Masui, D.; Tachibana, H.; Inoue, H.; Takagi, S. Efficient Excited Energy Transfer Reaction in Clay/Porphyrin Complex toward an Artificial Light-Harvesting System. *J. Am. Chem. Soc.* **2011**, *133* (36), 14280–14286. https://doi.org/10.1021/ja204425u.

(7) Wu, D.; Huang, Y.; Hu, S.; Yi, X.; Wang, J. Sensitive Hg2+ Sensing via Quenching the Fluorescence of the Complex between Polythymine and 5,10,15,20-Tetrakis(N-Methyl-4-Pyridyl) Porphyrin (TMPyP). *Sensors (Switzerland)* **2018**, *18* (11). https://doi.org/10.3390/s18113998.

(8) Han, Y.; Lupitskyy, R.; Chou, T. M.; Stafford, C. M.; Du, H.; Sukhishvili, S. Effect of Oxidation on Surface-Enhanced Raman Scattering Activity of Silver Nanoparticles: A Quantitative Correlation. *Anal. Chem.* **2011**, *83* (15), 5873–5880. https://doi.org/10.1021/ac2005839.

(9) Krivenkov, V.; Rakovich, Y. P.; Samokhvalov, P.; Nabiev, I. Synergy of Excitation Enhancement and the Purcell Effect for Strong Photoluminescence Enhancement in a Thin-Film Hybrid Structure Based on Quantum Dots and Plasmon Nanoparticles. *J. Phys. Chem. Lett.* **2020**, *11* (19), 8018–8025. https://doi.org/10.1021/acs.jpclett.0c02296.
